# Ergonomic Insect Headgear and Abdominal Buckle with Surface Stimulators Manufactured via Multimaterial 3D Printing: Snap-and-Secure Installation of Noninvasive Sensory Stimulators for Cyborg Insects

**DOI:** 10.34133/cbsystems.0406

**Published:** 2025-09-22

**Authors:** Phuoc Thanh Tran-Ngoc, Kewei Song, Thu Ha Tran, Kazuki Kai, Qifeng Lin, Hirotaka Sato

**Affiliations:** School of Mechanical and Aerospace Engineering, Nanyang Technological University, Singapore 639798, Singapore.

## Abstract

Insects have been integrated with electronic systems to create cyborg insects for various practical applications by utilizing their inherent adaptability and mobility. Nevertheless, most cyborg insects’ preparation depends on the invasive method, which can cause harm to critical sensory organs and restrict the obstacle-negotiating capabilities of cyborg insects. We present wearable devices with headgear and abdominal buckle that address these challenges using hooking mechanisms, multimaterial 3-dimensional printing, and selective electroless plating. These devices attach securely to the antenna scape and abdominal tergum without damaging functional organs, thereby preserving the insect’s natural sensory functions and physical intactness. Besides, the electrodes attach and detach easily without using adhesives, reducing the time required for cyborg insect preparation and enabling the reuse of insects. Experiments show that cyborg insects with wearable devices spend less time traversing obstacles than those prepared using invasive methods. Additionally, the potential for practical navigation tasks is further demonstrated by the cyborg insect’s capacity to navigate along the “S”-path. This work advances scalable, efficient, and ethical utilization of cyborg insects in the fields of robotics and biohybrid systems.

## Introduction

Cyborg insects, which integrate biological organisms with electronic systems, provide a revolutionary platform in robotics, providing distinctive functionalities for applications, including search and rescue, environmental monitoring, and surveillance [1–3]. Unlike centimeter-sized robots that are composed entirely of mechanical structures, cyborg insects retain their natural mobility and ability to adapt to complex terrain. This enables them to operate efficiently in confined environments like postdisaster urban spaces and pipelines, thanks to their natural mobility, low energy consumption, and environmental adaptability. Cyborg insects thus hold numerous advantages over conventional robotic systems, which are often energy-intensive and exhibit limited ability to traverse hard-to-reach terrains [4,5]. Thus, inhibition of cyborg insect movement is commonly accomplished via electrostimulation of their musculature, nervous systems, and sensory organs [6–9].

The extreme load-bearing capacity (15 g) of the Madagascar hissing cockroach has made it one of the most commonly used models for cyborg insect research [10–12]. Efforts to enhance the natural abilities and mobility of cyborg insects have included several innovations. For example, flexible solar cell modules have been implemented to overcome the power limitations, offer a sustainable power source without interfering with mobility, and allow long life operation of on-board electronics [13]. Inspired by ladybugs’ ability to right themselves, 3-dimensional (3D)-printed artificial limbs were developed that enable Madagascar hissing cockroaches to stand upright once they had been flipped over, allowing them to cross complicated terrain [14]. Besides, a dual-mode, 3-wheeled exoskeleton has been developed to be mounted on the cyborg insect. This device will enhance cyborg insects’ load capacity and locomotion control precision, enabling them to carry advanced sensors and perform complex tasks [15]. Another example is the strategic design of the cyborg cockroach, which integrates a control panel and an implanted battery. Advancing mobility performance in limited spaces can be achieved through the reduction of physical limitations by improving success rates while overcoming distances [16].

**Fig. 1.**
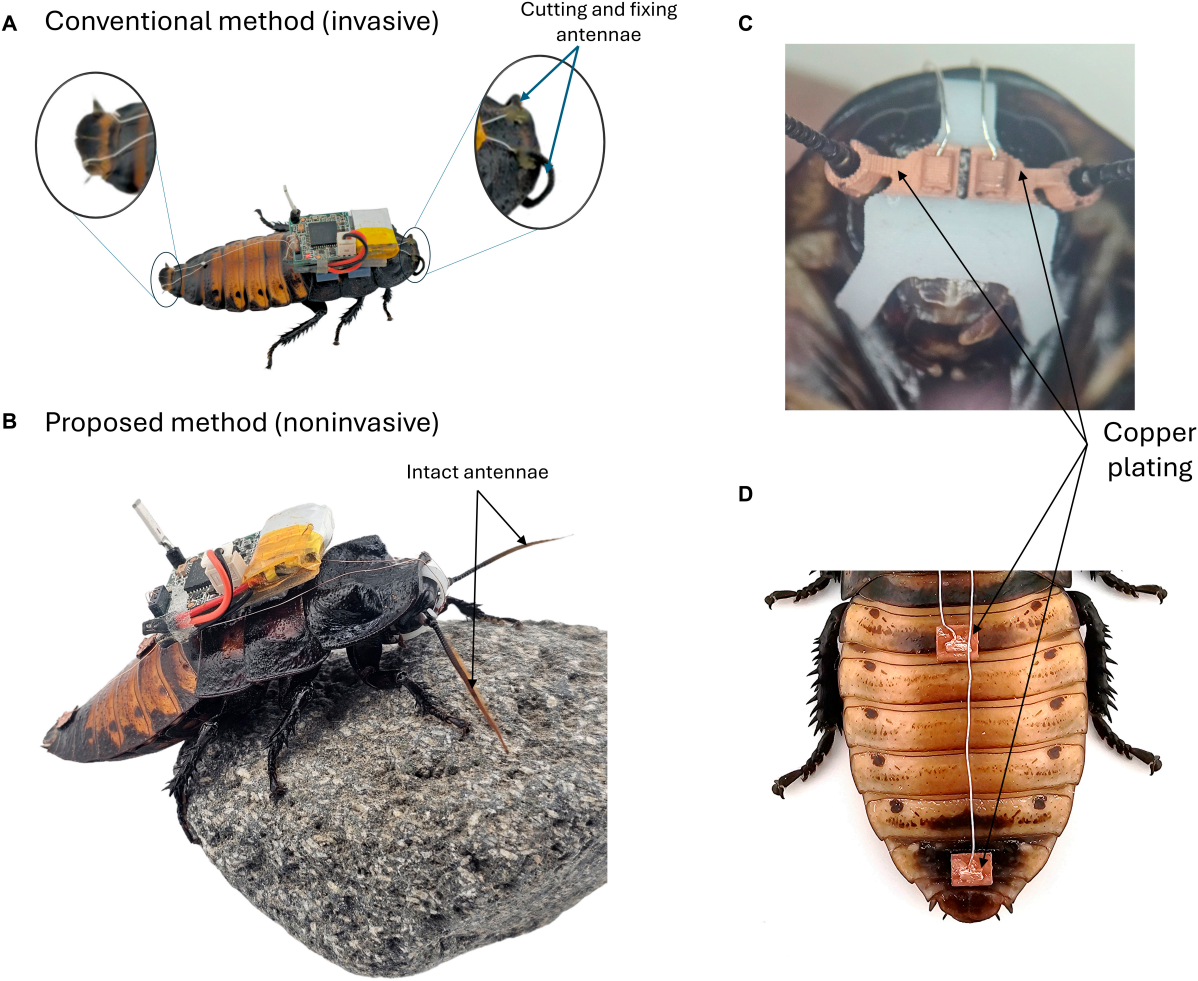
Wearable devices using noninvasive electrodes with metal plating for cyborg insect preparation. (A) Conventional method of attaching electrodes: The antennae and cerci are cut using scissors, and an insect pin punctures the abdominal segment. This process is to create open spaces for inserting the electrodes inside these parts. These methods cause irreversible damage to the insect’s sensory organs. (B) The proposed preparation utilizes wearable devices with noninvasive electrodes and metal plating. For the antennae, a wearable device plays as the headgear that will grasp the scape to transfer the electrical stimuli without inserting the electrode. Thus, it preserves the sensory function of the antennae to sense and navigate obstacles. Similarly, the electrodes implanted into the cerci and second abdominal segments were replaced by the wearable device. This device functions as an abdominal buckle that attaches firmly to the sixth and second segments. Therefore, these wearable devices avoid physiological harm and allow for the reuse of the insect. (C) The headgear designed for attachment to the cockroach’s head includes 2 connectors that securely grasp the scape of the antennae. These connectors are plated with copper and are connected to the electrical board using silver wires. (D) The abdominal buckle, intended for attachment to the second and sixth abdominal segments, is entirely plated in copper, thereby enhancing the conduction of electrical signals from the electrical board via silver wiring.

Electrical stimulation using implanted wires is a method of choice when preparing cyborg insects because of its ease of installation and control of stimulation intensity, but it also has many inconvenient drawbacks. Such implants being invasive harm the working organs, like antennae and cerci, which alter the physiology of the insect (Fig. 1A) [17]. Given that antennae are primary olfactory organs and that they play an important role in detecting obstacles, damage may drastically affect insect perception of the environment and diminish their capacity to execute natural self-avoidance behaviors [18,19].

Beyond practical considerations, there is an urgent ethical dimension to invasive synthetic insect methods. Cutting off antennae or cerci is a traditional method that raises significant concerns about animal welfare and is consistent with broader discussions on humane research practices. The “3Rs” framework (replacement, reduction, and refinement) introduced by Russell and Burch captures these concerns by emphasizing the necessity of minimizing injury and maximizing animal reuse whenever feasible [20,21].

In order to address this issue, a noninvasive approach was implemented to replace implanted electrodes with surface electrode by resilient conductive film [12]. These noninvasive electrodes were wrapped around the antennae basement and adhered to the abdominal segment of the cockroach to prepare the cyborg insect. Thus, this approach eliminates the antennae-cutting and maintains the cyborg insects’ navigation ability. However, these films often relied on poly ionic liquid (PIL) gels or similar adhesives to secure electrodes onto the insects’ smooth exoskeleton, as demonstrated by Lin et al. [12]. This approach might encounter a variety of obstacles. Initially, the adhesives may become weak or degrade over time, resulting in a compromised electrical connection due to the insect’s movement. Secondly, the application and removal of gels can be time-consuming and may cause partial harm to the exoskeleton during detachment. Third, the maintenance of stable contact with curved or malleable body parts necessitates meticulous preparation and skill. Consequently, despite the fact that solely adhesive-based methods are less invasive than cutting the cerci or antennae, they can still restrict long-term stability [12].

For both invasive and noninvasive methods to prepare cyborg insects, there are many steps with specific equipment, such as a microscope, handheld soldering tool, and adhesives like beeswax or PIL gel [2,3,12,22,23]. These procedures are time-consuming and require significant skill. Furthermore, invasive methods limit the insect to a single use due to the cutting of the antennae and cerci. Thus, it is necessary to design the wearable device with hooking mechanisms that allow the electrodes to attach firmly and detach easily from the insect’s antennae and abdominal segments. This innovation improves both preparation time and reusability of cyborg insects.

The cockroach’s antenna comprises 3 parts: the scape, the pedicel, and the flagellum. The scape is closest to the head, the pedicel occupies the intermediate position, and the flagellum is the distal and most flexible segment [24,25]. The scape is a robust and slightly elongated structure that connects to the cockroach’s head at the antennal socket. The pedicel is smaller than the scape and holds the Johnston organ, which senses the flagellum’s airflow, vibrations, and relative positioning. The flagellum comprises many tiny flagellomeres with arrays of sensory receptors for detecting environmental cues. Based on antenna anatomy, the scape is an ideal choice for contacting the wearable device because of its sturdy and large structure, stable connection to the head, and avoidance of disrupting the sensory functions of the pedicel and flagellum.

Advances in manufacturing methods have enabled the development of wearable devices that can attach to insect antennae and transmit electrical stimuli. Traditional manufacturing methods have difficulty producing small components, handling complex surface geometries, and including many materials in a single part. New technologies such as digital light processing (DLP)-based multimaterial 3D printing and selective electroless plating have been introduced to solve these issues [26,27]. DLP 3D printing of multimaterial enables the fabrication of wearable devices consisting of 2 different materials: an active precursor resin to manufacture the elements contacting the insect’s body that will be plated conductive metal and a normal resin to print the nonconductive structural parts of the device. The selective electroless plating process coats a thin metal layer onto the surfaces made by the active precursor resin. Thus, these plating components will transmit electrical stimuli from the circuit board to the insect’s sensory organ for navigation functions [27].

Here, we report wearable devices, such as headgear and abdominal buckles with surface stimulators, designed for cyborg insect preparation and navigation. The ergonomic design of our wearable device allows it to conform naturally to the insect’s anatomy, ensuring a secure and comfortable fit without adhesives or invasive methods. This approach preserves the insect’s natural movement and sensory function, which are key for effective navigation in complex environments. These devices had advanced hook mechanism design structures, multimaterial DLP 3D printing, and selective electroless plating methods to overcome the existing limitations (Fig. 1B to D). Thus, it streamlines the preparation process and allows insects to be reused.

## Materials and Methods

### Preparation of cyborg insects

We employed the reassembled cyborg insects reported in Ref. [11]. We chose Madagascar hissing cockroaches (*Gromphadorhina portentosa*, ~6 g, 6 cm, hereafter cockroach) as our insect model (Fig. 1B). Cockroaches were in NexGen Mouse 500 terrarium from Allen Town and were fed sliced carrots weekly. The environmental conditions were maintained at 25 °C and 60% relative humidity. Research involving cockroaches was approved by the National Environmental Agency (Permit number NEA/PH/CLB/19-00012).

Control of locomotion was established using electrical stimulation of the cockroach’s antennae and the sixth abdominal segment. Antenna stimulation caused the cockroach to turn in the opposite direction, while stimulation of the sixth abdominal segment induced acceleration. The electrical circuit backpack used in this system was adapted from previous research [11].

**Fig. 2.**
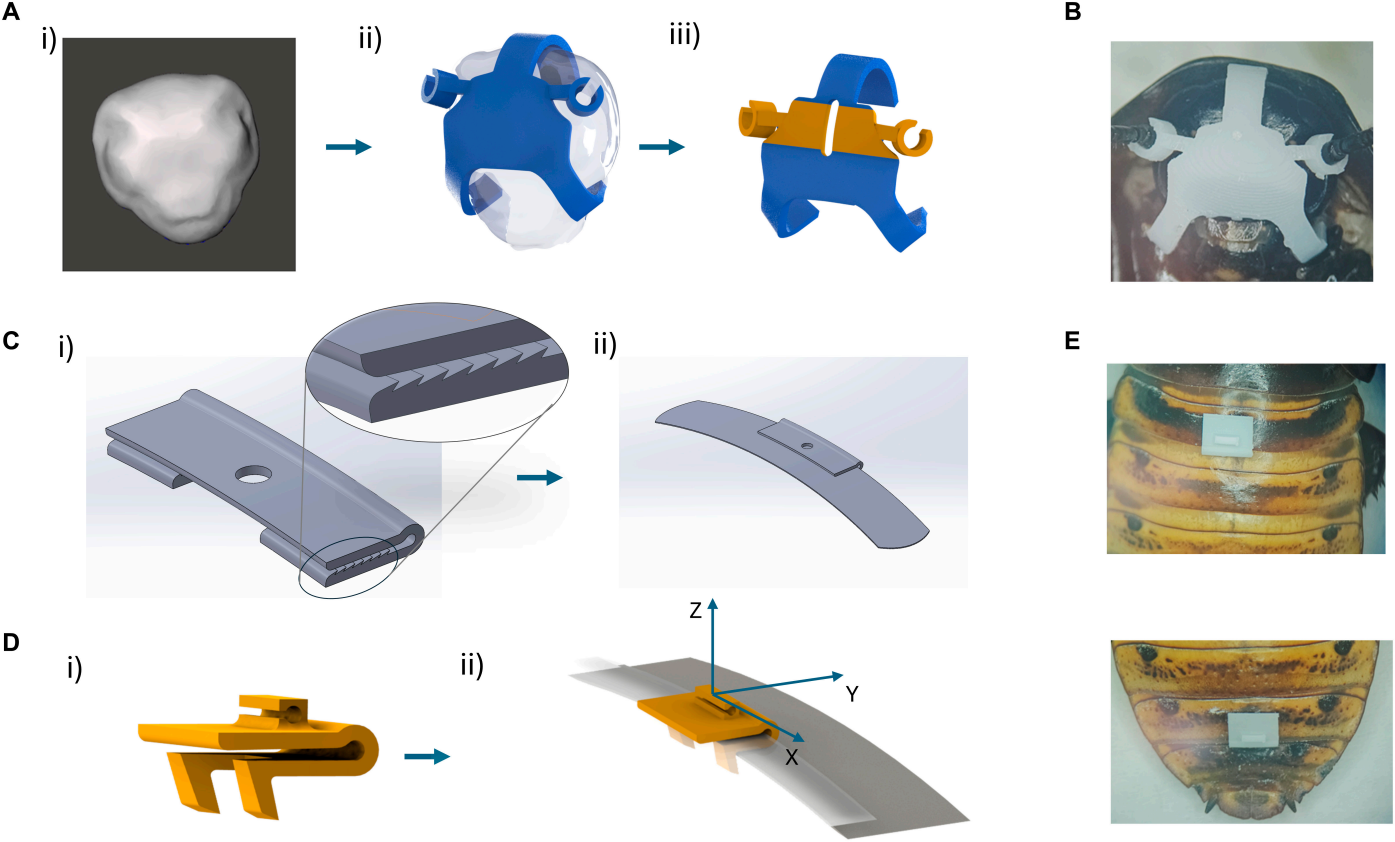
Designing the wearable device using the 3D model of the cockroach head and abdomen segments. (A) Wearable device for the antennae: (i) A 3D model of the cockroach’s head was used as a reference to design the wearable device for the antennae. (ii) Two hooking mechanisms were incorporated to ensure the device attaches firmly to the cockroach’s head while also providing a secure connection at the antennae’s scape. The prototype was manufactured using a 3D printing method and tested for its attachment capability on a live cockroach. (iii) The improved design with 2 types of materials: the blue part is made with normal resin, the green part is made with active precursor resin that will be plated to conduct electrical stimuli, and a groove was added to separate the left and right parts of the antennae. (B) The prototype of wearable device was manufactured using a 3D printing method and was mounted on the head of the cockroach with the 2 connectors attached to the antennae. (C) The first design of the wearable device for the abdomen segments: (i) This design uses a hooking mechanism with pointed edges that are angled for easy attachment at the edges of the cockroach’s abdomen segment. (ii) The wearable device can firmly attach to the segment because pressure force is created because the gap between the upper and lower parts of the hooking mechanism is smaller than the thickness of the abdomen segment. (D) The improved design of the wearable device for the abdomen segments: (i) We modified the hooking mechanism to utilize hooks that clamp onto the edge of the tergum rather than relying on friction from pointed edges. (ii) This design integrates 3 main functions—the U-shaped clamp, the overlapping structure of the tergum, and the gripping hooks—which can cooperate to firmly fix the wearable device on the cockroach’s abdominal segment. (E) The prototype was manufactured using a 3D printing method and was attached to the second and sixth abdominal segments of the cockroach.

**Fig. 3.**
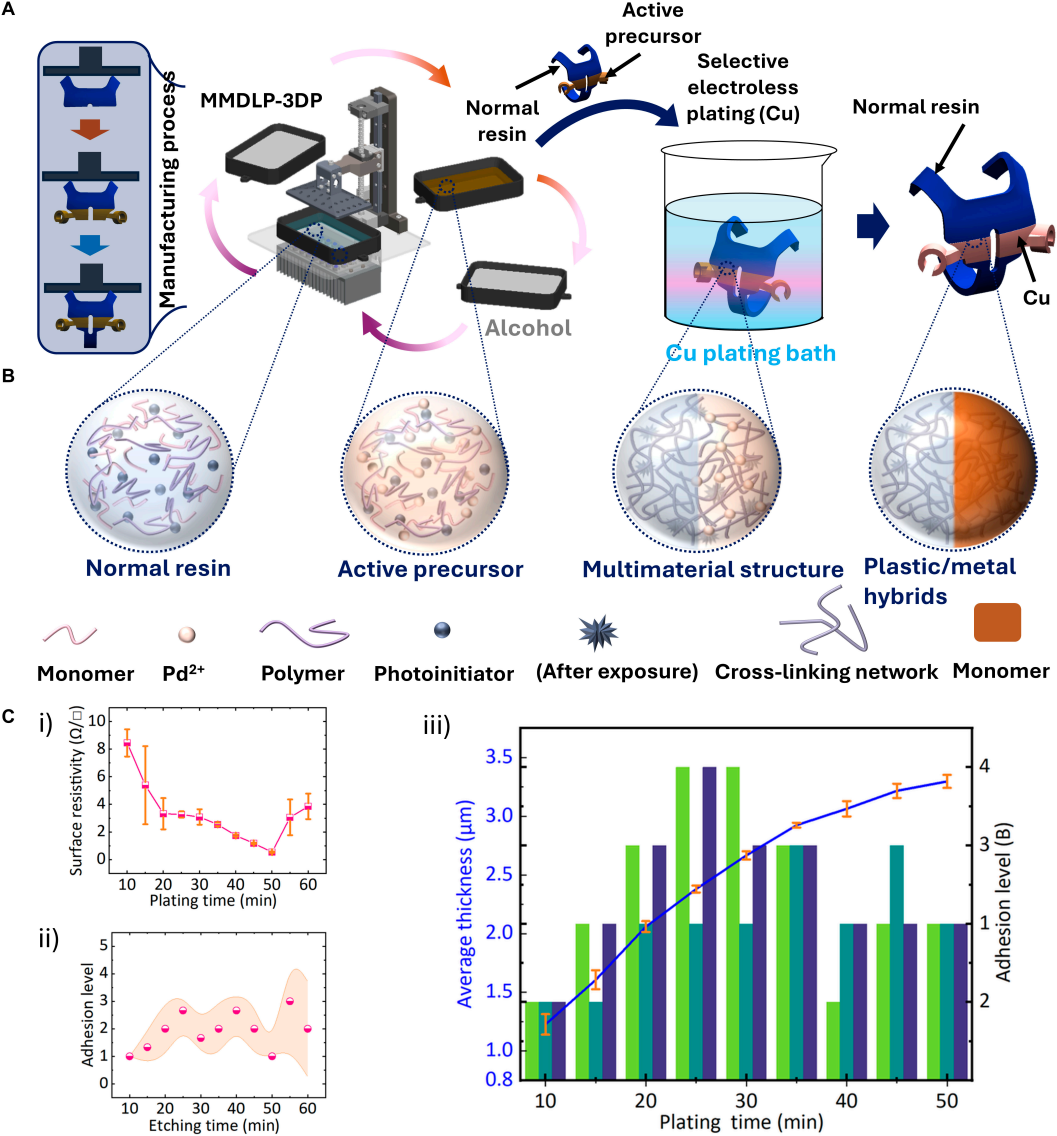
Conceptual framework to produce wearable devices utilizing multimaterial DLP 3D printing technology, selective electroless plating technology based on active precursors, and its mechanical and electrical characteristics. (A) Schematic representation of the multimaterial DLP 3D printing (MMDLP-3DP) technique followed by selective electroless plating to produce plastic/metal hybrid structures. The process begins with the 3D printing of both normal resin and active precursor resin containing Pd^2+^ ions. After printing, the structure is developed in alcohol to remove any excess resin. The active precursor is then selectively activated for metallization in a Cu electroless plating bath, forming conductive metal regions while the normal resin remains nonmetalized. (B) Depiction of the chemical composition at different stages: normal resin (without Pd^2+^), active precursor (with Pd^2+^), multimaterial printed structure after cross-linking via UV exposure, and the final plastic/metal hybrid structure post-Cu plating. (C) Characterization results: (i) Surface resistivity as a function of plating time, showing decreased resistivity with longer plating duration. (ii) Coating thickness over plating time, showing the progressive thickening of the metal layer as plating time increases. (iii) Combined characterization of average coating thickness (bars) and adhesion level (line plot) over various plating times. Each plating duration is represented by 3 bars, with each bar corresponding to 1 of 3 independent measurements. Adhesion level was evaluated using the ASTM D3359-09 tape test (Rating B) and is plotted with mean values and standard deviation. The blue line depicts a gradual increase in plating thickness with extended deposition time, reaching approximately 3.4 μm at 50 min.

Cockroaches were anesthetized by exposing them to CO₂ in a sealed container for 30 s. For the antennae, the 2 lower hooks of the wearable device were first placed beneath the head. The top hook was gently placed using tweezers at the top of the head, where the elastic material allowed it to stretch and temporarily deform to fit securely along the sides and partially onto the back of the head (Fig. 2A and S1A). When inserting the hooks, the antenna was aligned with the gap in the C-shaped connector and pressed into this. The C-shaped connector has elastic properties, so they can deform somewhat as the antenna gets inserted to ensure a tight fit. We did the same for the second antenna (Fig. 2B and S1B). The inner surface of the C-shaped connector was coated with conductive paste (Spectra360, Parker Laboratories, USA) to ensure that the inner wall of the C-shaped connector contacted the outer surface of the antennae’s scape. As the scape of the antennae was mounted into the connector that was plated with copper (Fig. 3A), this paste ensured a consistent path for the conduction of electrical stimulation to the antennae.

The second abdominal segment was specified for grounding. The bottom hooks of the wearable device were inserted into the spaces between the first and second abdominal segments and the sixth and seventh abdominal segments until the hooks firmly clutched the edges of the tergum (Fig. 2D and E and Fig. S1B).

Electrical stimulation was used to control the cockroach’s movements. Currents were applied to the antenna electrodes, and ground-induced turns in the opposite direction were triggered, while currents through the cerci and ground triggered acceleration. A biphasic square waveform with a frequency of 42 Hz, a 50% duty cycle, and voltages ranging from 2.0 to 3.5 V was employed for stimulation.

### Neural recording during stimulation

#### Dissection

To evaluate the response to electrical stimuli and determine the optimal stimulation strength, neural activity was recorded from cockroaches. The ventral nerve cord (VNC), a pair of nerve bundles that interconnect the brain and thoracic and abdominal ganglia, was chosen as the recording site for its accessibility and importance to locomotion control of insects [28,29]. Cockroaches were subjected to CO₂ anesthesia for immobilization and were equipped with the wearable devices to deliver electrical stimulation to the antenna and the abdomen. Metal pins were used to fix the animals onto a rubber block with the ventral side up. The cuticle was cut by a razor blade, and internal tissues (fat body, trachea, and muscle) were removed to expose the VNC at the neck or fourth abdominal segment for antenna or sixth segment stimulation, respectively. Cockroach saline was used to rinse the VNC to allow for visibility under the microscope.

#### Neural recording with a suction electrode

We used a glass pipette suction electrode to extracellularly record the neural signal from the VNC. A glass capillary (2.0 mm in outer diameter) was heated with flame and pulled to make a pipette. The tip of the pipette was cut and flamed to be blunt so that the internal diameter of the pipette was slightly smaller than the VNC. The glass pipette was filled with cockroach saline before each recording session. The pipette was attached to an electrode holder (ESW-M20P, Warner Instruments) which was connected to a syringe through a polyethylene tube and a 3-way stopcock. Ag-AgCl wires (0.2 mm in diameter) were used as recording and reference electrodes. The recording electrode was inserted into the glass pipette through the electrode holder, whereas the reference electrode was placed outside of the pipette near the tip. To obtain neural signal from the VNC, either side of the VNC connector was cut by a fine microscissors and the cut end close to the stimulation site (antenna or sixth abdominal segment) was suctioned into the pipette by applying negative pressure to the tube using the syringe. The tight contact between the pipette and the VNC allowed the isolation of the electrical (neural) signal from the surrounding tissue. The previous study on neuroanatomy of Madagascar hissing cockroach revealed a neural pathway from the deutocerebrum (antenna sensory-motor center) in one side of the brain to the thoracic ganglia on the other side [30]. Therefore, the stimulation was applied to the antenna contralateral to the recording site on the VNC. For sixth segment stimulation, recording was made on either side of the VNC because the wearable devices were attached in the middle of the body. The neural signal was analog-filtered (band-pass filter, 100 Hz to 10 kHz), amplified using a differential amplifier (Model 3000, AM Systems), and sampled at 20 kHz with an A/D converter (PowerLab 26T, AD Instruments).

#### Stimulation

**Fig. 4.**
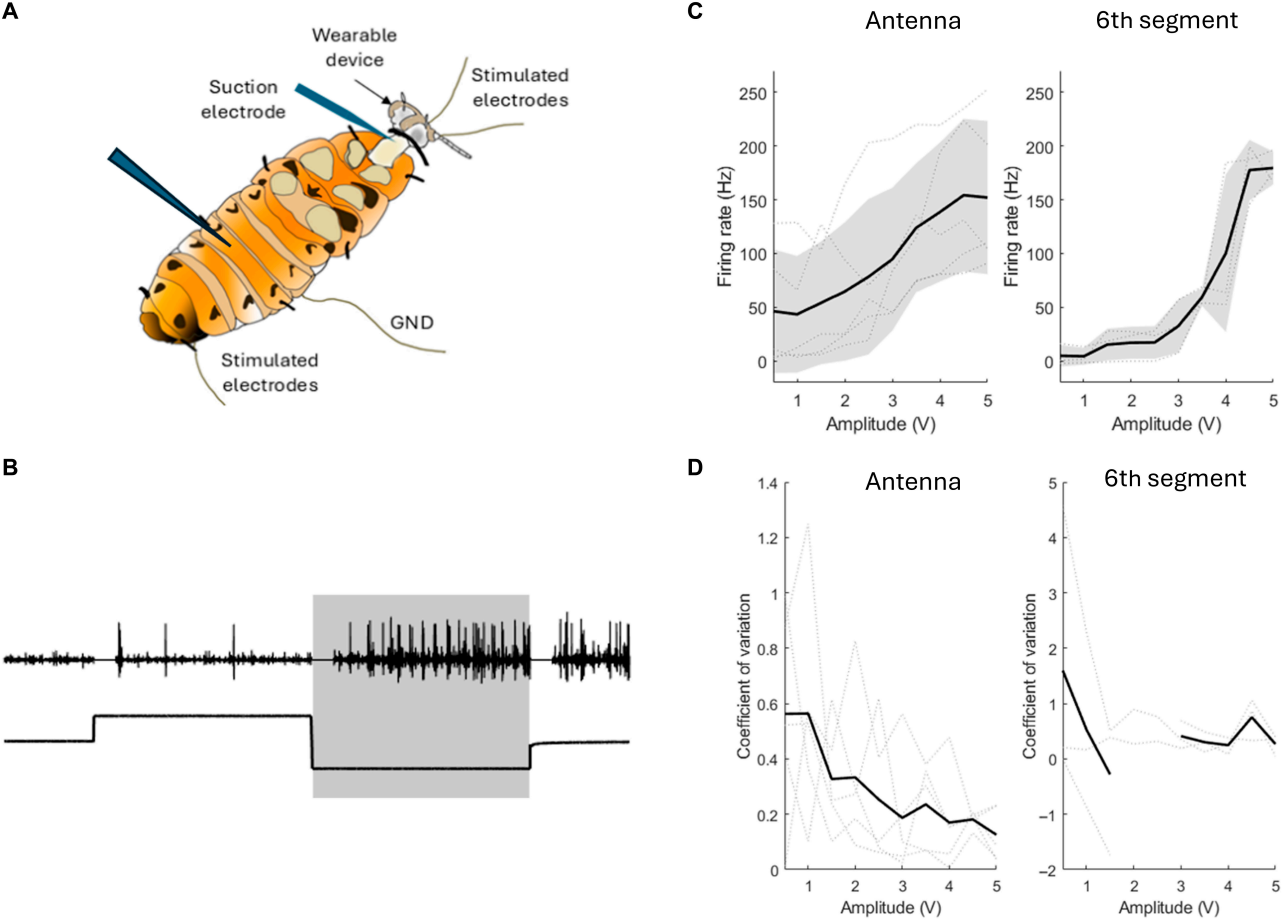
Neural response to antenna stimulation and sixth abdominal segment stimulation using noninvasive electrodes with metal plating. (A) Experiment setup: The insect was secured on a platform and equipped with headgear at the antennae, and abdominal buckles at the second and sixth abdominal segments. Neural recordings from the ventral nerve cord were conducted to observe the insect’s reaction to electrical stimulation in 2 cases: stimulation at the antennae and stimulation at the sixth abdominal segment. The second segment was used as the ground (GND). (B) Neural activity and antenna stimulation: Black dots represent detected neural spikes. Neural reaction spikes within the shaded area were further analyzed to assess response patterns. (C) Firing rate responses across varying stimulation amplitudes (0.5 to 5 V), with dotted lines showing individual trials and the solid black line representing the average firing rate across animals. Shaded regions denote ±1 standard deviation. (D) Coefficient of variation (CV) of the firing rate across amplitudes, indicating the consistency of the neural response.

Electrical stimulation was delivered via the wearable devices attached to the antenna and abdominal segment of the cockroaches. Wearable device played as ground was attached to the second abdomen (Fig. 4A). We deployed charge-balanced bipolar pulse to avoid habituated response [17]. The backpack microcontroller delivered a single bipolar square-wave pulse (positive–negative pulse) at 1 Hz with a duty cycle of 50%. Because the wearable device attached to the second abdominal segment served as the ground electrode, the stimulation site (antenna and sixth abdominal segment) received the positive pulse first. Stimulus amplitude varied from 0.5 to 5.0 V with 0.5-V steps. Neural signal was monitored during experiment and stimulation was applied when phasic neuronal activity was not observed. Each condition was replicated 3 times with at least an interval of 5 s.

#### Analysis of neural signal

Neural signal included artifacts derived from the stimulation, which affected digital filtering of the signal necessary for removal of noise. To mitigate this, 50 ms of neural data, beginning at 0, 0.5, and 1.0 s (the pulse edges), were set to zeros (Fig. 4 and Fig. S3). The remaining neural signals were filtered using a second-order Butterworth filter with a passband of 300 to 3,000 Hz. The electrode placed at the end of the nerve cord records a biphasic waveform (positive–negative peak) of action potential traveling along the axon (Fig. S3) [31]. To detect neural spikes reliably, we chose the positive or negative peak depending on the averaged magnitude of peak, whichever is larger.

Neural spikes were detected using a threshold defined in Eq. (1) [32]:

$$T=5\times \text{median}\left(\frac{\left|x\right|}{0.6745}\right)$$ (1)

where *x* is considered as a filtered signal. Due to the difference in ion concentration across the membrane of a neuron, the inside is charged negatively and the outside is charged positively. As depolarization of membrane potential elicits an action potential to neuron, the number of spikes during negative pulse was analyzed in a further step. The firing rate (number of spikes per second) was calculated using Eq. (2):

$$\text{firing rate}=\frac{N-N_{0}}{\left(W-50\right)/1,000}$$ (2)

where *W* is the width of the negative pulse in milliseconds, *N* is the number of spikes during the pulse, and *N*_0_ is the number of spikes within the same time window (*W* − 50) before stimulation. Coefficient of variation (CV; ratio of the standard deviation [SD] to the mean) was also calculated based on the spike events. Using this method, neural responses to a range of stimulation voltages could be determined, which could be used to evaluate the effect of the applied stimulation. All analysis was conducted using MATLAB (MathWorks).

### Locomotion control of cyborg insects

**Fig. 5.**
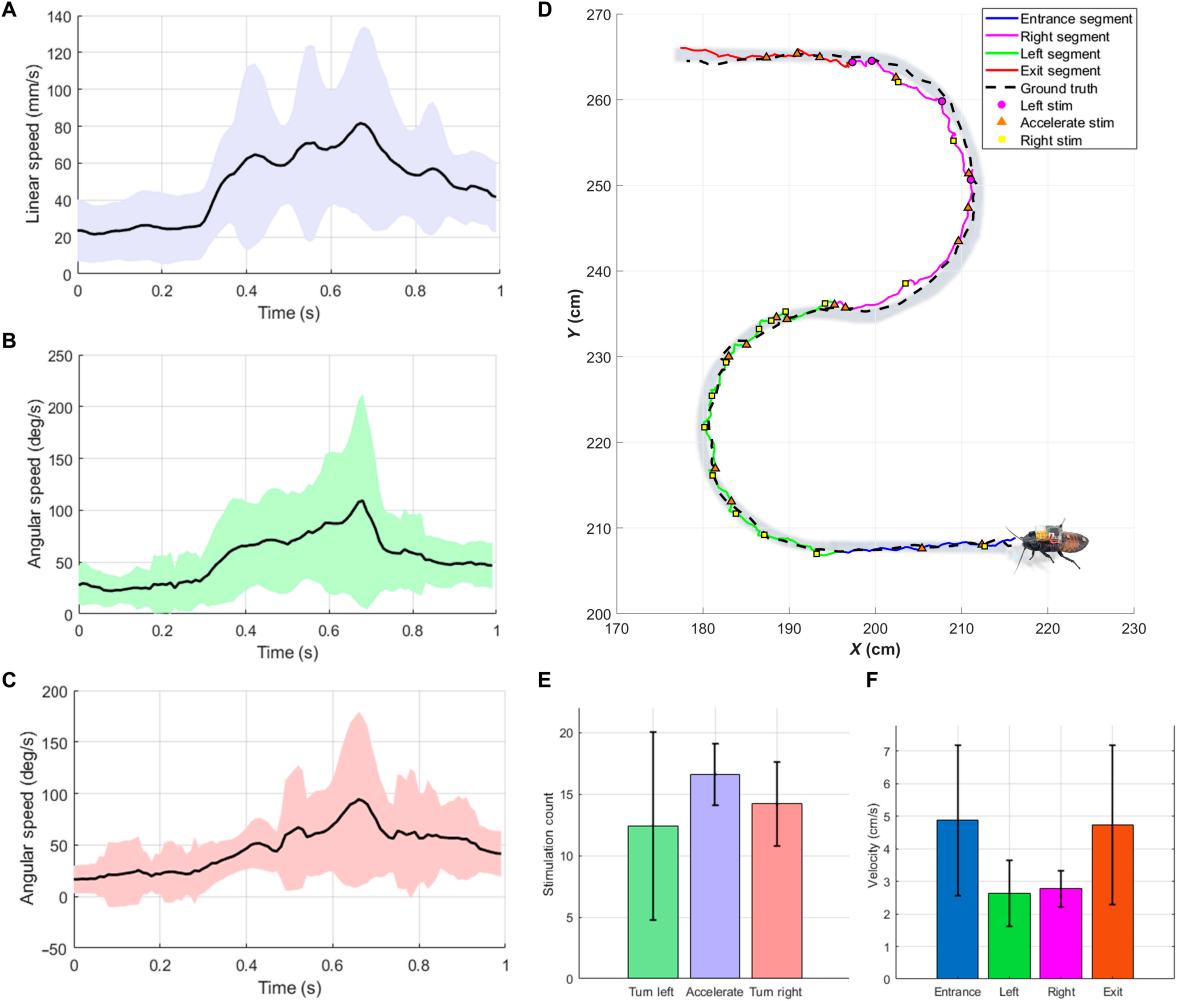
Insect’s motion induced by electrical stimulation using noninvasive electrodes with metal plating. (A) Acceleration stimulus (induced linear speed: mean = 23.81 mm/s [before stimulation] and 56.78 mm/s [during stimulation]; SD = 15.75 mm/s [before stimulation] and 20.86 mm/s [during stimulation]). (B) Left-turning stimulus (induced angular speed: mean = 25.36°/s [before stimulation] and 64.95°/s [during stimulation]; SD = 13.86°/s [before stimulation] and 33.43°/s [during stimulation]). (C) Right-turning stimulus (induced angular speed: mean = 20.41°/s [before stimulation] and 52.96°/s [during stimulation]; SD = 19.35°/s [before stimulation] and 22.79°/s [during stimulation]). (D) “S”-path navigation trial: The cyborg insect was manually controlled along a predefined “S”-shaped path (ground-truth length: 142.95 cm) using headgear and abdominal buckle. The actual trajectory (colored by segment: entrance = blue, right turn = magenta, left turn = green, exit = red) closely followed the designed path (black dashed line). Stimulation points are shown with markers: left (magenta circles), acceleration (orange triangles), and right (yellow squares). Quantitative analysis of this trial yielded an RMSE of 1.80 cm, a total travel distance of 157.09 cm, and a duration of 52.11 s, highlighting the accuracy and control stability of the system. (E) Mean ± standard deviation of stimulation counts for turn left, acceleration, and turn right commands, computed across 5 independent trials. (F) Mean ± standard deviation of cyborg velocity for each of the 4 path segments (entrance, left turn, right turn, and exit), computed across 5 trials.

**Fig. 6.**
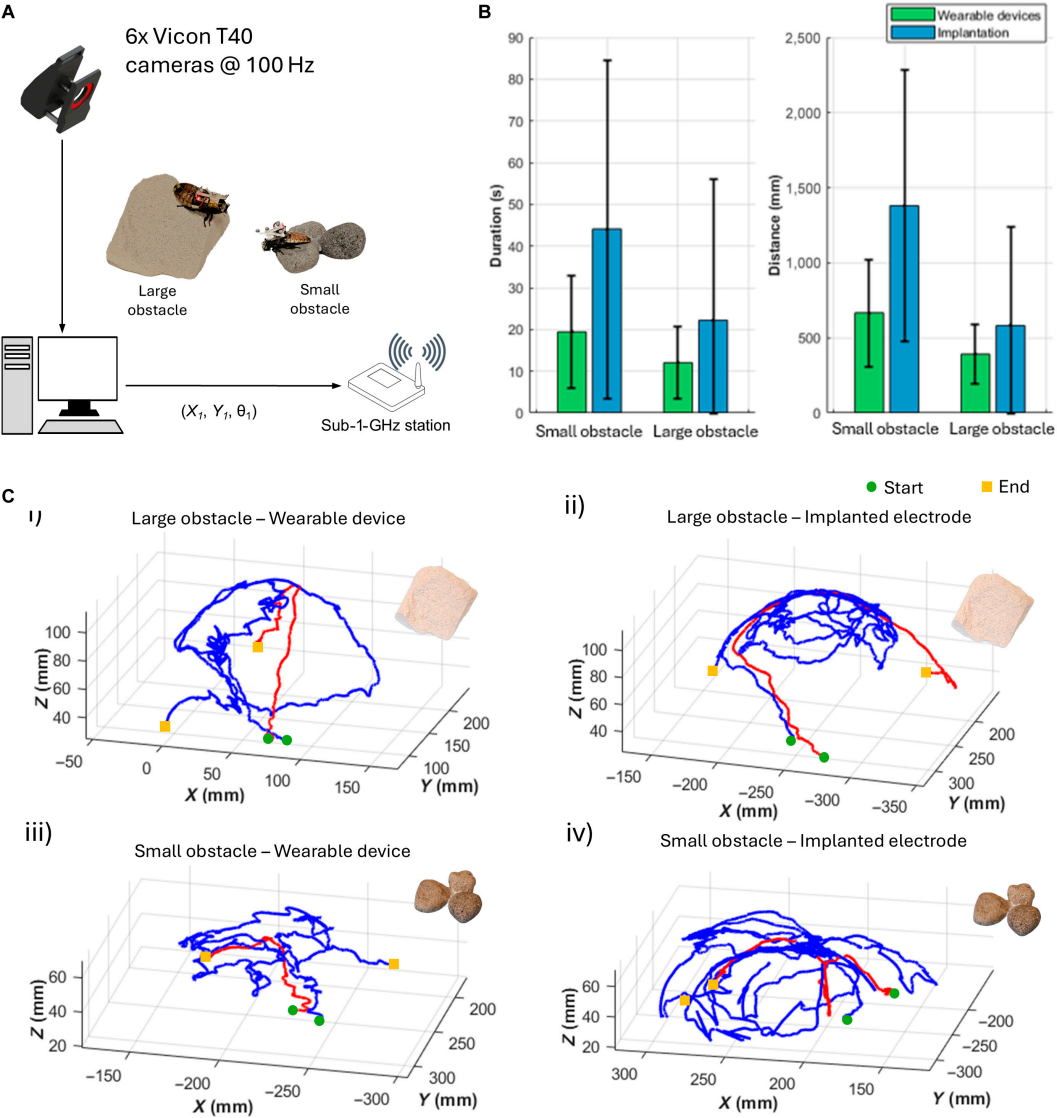
Experimental results demonstrating the advantage of the wearable device compared to the implanted electrode. (A) Motion tracking system for the cyborg insect’s locomotion control. The VICON camera system provides feedback on the insect’s position to the workstation, which sends stimulation signals to the cyborg insect through a central board. (B) The mean and standard deviation of duration and distance were compared across the wearable device and implantation methods for both large and small obstacles, highlighting the performance differences between the 2 approaches (*N* = 3 insects, *n* = 30 trials). (C) The positions of the cyborg insects while climbing over obstacles were tracked, and their trajectories were plotted for the 2 trials with the quickest and slowest durations in each of the following scenarios: (i) cyborg insect with a wearable device climbing over a large obstacle, (ii) cyborg insect with an implanted electrode climbing over a large obstacle, (iii) cyborg insect with a wearable device climbing over a small obstacle, and (iv) cyborg insect with an implanted electrode climbing over a small obstacle. The green circle and yellow square denote the start and end points of each trajectory, respectively.

Three cyborg insects (*N* = 3) were tested for locomotion control, including turning left, right, and accelerating (Fig. 5A to C). Each stimulation type was applied 5 times to each insect using a bipolar pulse wave (0.6 s duration, 42 Hz frequency, with voltages ranging from 2.0 to 3.0 V). During the experiment, a motion tracking system (VICON) was employed to monitor the 2 markers attached to the insect’s body (Fig. 6A). These markers captured the cyborg insects’ reactions to the stimulation, such as changes in turning direction and acceleration before and after stimulation.

To study the feasibility of control, we analyzed the linear and angular velocities of the insects during stimulation. Every trial was 1,000 ms long: 200 ms before the stimulation, 600 ms during stimulation, and 200 ms after stimulation. To evaluate performance and variation, the average and SD of the velocities were calculated per trial.

To assess the wearable devices’ navigation abilities, a gamepad manually controlled one cyborg insect along an S-shaped path with a length of around 1 m. The gamepad created control signals like “turn left”, “turn right”, and “move forward” and sent them to the central board, which then transmitted the control signals to the backpack mounted on the cyborg insect via sub-1-GHz communication.

### Impedance measurement of wearable electrodes

To assess the stability and suitability of electrical contact, we measured the impedance between the wearable device and the cockroach body at 1 kHz (*N* = 3 cyborg insects). Each cyborg is equipped with a headgear (for antenna stimulation) and 2 abdominal buckles (for acceleration and grounding).

Measurements were taken at the following electrode paths:

• Left antenna to second abdominal segment.
• Right antenna to second abdominal segment.
• Sixth abdominal segment to second abdominal segment.

For antenna electrodes, a thin layer of conductive gel was applied between the scape surface and the inner wall of the connector of the headgear. This gel will improve electrical conductivity while preserving the mechanical stability of the device.

### Manually controlling the cyborg insect along the S-path

To evaluate the navigational ability of the cyborg insect with wearable devices, the insect was connected to an electrical board that provided stimulation signals for turning and acceleration. A Bluetooth gamepad was paired with a custom personal computer (PC)-based graphical user interface to manually trigger stimulation commands. These commands were sent wirelessly from the PC to the electrical board by sub-1-GHz communication [11]. Then, electrical stimuli were delivered to the insect for directional control.

The cyborg was guided along a predefined S-shaped path consisting of 4 segments: a straight entrance segment, a half-circle curve to the left, a half-circle curve to the right, and a straight exit segment (Fig. 5D). The total path length was approximately 1.4 m. The operator monitored the cyborg insect’s position in real time to check its position with the S-path. Then, the turning or acceleration commands were manually triggered to control the cyborg along the S-path.

A reflective marker set was attached to the cyborg insect, allowing its position to be tracked by a VICON tracking system. During each trial, both the stimulation events and positional data with time stamps were recorded and saved on the PC. A total of 5 trials (*N* = 3, *n* = 5) were conducted using 3 cyborg insects. In order to obtain the ground-truth position for the S-path, a marker was manually moved along the path and its trajectory was recorded during the movement.

### Comparison of obstacle negotiation between wearable devices and the conventional implantation method

These wearable devices allow the cyborg insects to maintain antenna mobility compared to the conventional implantation method, which severs the antennae and attaches them to the pronotum. To investigate the performance in these 2 states, an experiment was conducted with 2 kinds of obstacles: large obstacles, such as concrete blocks, and small ones, such as rocks (Fig. 6A). The small obstacles consisted of 3 rocks placed close together, each approximately 7 × 7 × 5 cm^3^. The large obstacle was a concrete piece with dimensions of approximately 10 × 10 × 15 cm^3^. Three cyborg insects were tested in each state, and each insect performed 10 trials (*N* = 3, *n* = 30).

The cyborg insects were positioned in front of the obstacles, where they were required to climb up, navigate the top surface, and climb down. During the experiment, an acceleration signal (0.4 s duration, 2.0 V) was automatically applied every 1 s to encourage the cyborg insects to remain active. A motion tracking system (VICON) monitored 2 markers attached to each insect’s body (Fig. 6A). These markers recorded the cyborg insects’ trajectories, traversal times, and distances traveled for each case.

### Material for 3D printer

PdCl_2_ nano-powder (purity: 99.0%) was procured from Kanto Chemical Company, Inc., while crystalline NH_4_Cl, used for dissolving PdCl_2_, was sourced from FUJIFILM Wako Pure Chemical Corporation. In addition, the chemicals needed to prepare the metal plating solution were also purchased from the Kanto Chemical Company, Inc. Ethanol (CH_3_CH_2_OH) for preparing the cleaning solution was purchased from Sigma-Aldrich Company, Inc., analytical pure (≥99%). The Art-Engineering photosensitive resin used for normal resin and required to prepare active precursor was purchased from JAMG HE Company, Inc., and the photosensitive wavelength is 395 to 405 nm.

### Preparation of functional ink

At room temperature (26 °C), each active precursor was prepared by dissolving 15.4 g of NH_4_Cl in 50 ml of deionized water, to which 270 mg of PdCl_2_ was added and dissolved with agitation. This yielded 50 ml of a saturated activation solution containing 0.2 wt% Pd^2+^. After allowing this solution to stand for a while, a 12-ml portion of the upper, clear part of the solution was used, with the undissolved portion used for the next preparation. Following that, 38 ml of normal resin was transferred to a vessel with a magnetic stirrer spinning at 1,000 revolutions per minute (RPM), and 12 ml of the activation solution was added dropwise. Following this addition, the mixture was stirred for another 30 min at 1,200 RPM to obtain 50 ml of an active precursor solution (the concentration of Pd^2+^ is approximately 0.058 wt%).

### Multimaterial DLP 3D printing

The implementation of the multimaterial 3D printing process is based on the principles of previous research [27] and is implemented in our upgraded multimaterial DLP 3D printing equipment. The specific process includes material switching, cleaning, and implementation. See Ref. [27] for detailed information. After the multimaterial parts are modeled and designed, the mechanical system and the control system are coupled to realize the multimaterial stacking manufacturing.

The required multimaterial components, consisting of normal resin serving as the substrate and an active precursor, form a part with the desired 3D topology. After setting the slicing layer thickness, single layer exposure time, number of bottom layers, bottom exposure time, and other process parameters in the slicing software on the PC, we can obtain the slicing dataset (containing all the required processing commands) for the different material topologies that make up the multimaterial parts. Then, the developed MM-DLP3DP will automatically complete the manufacturing of the parts according to the processing commands. In this process, the printer automatically switches materials and cleans between them to avoid cross-contamination. A 405-nm, 90-W ultraviolet parallel light source with a strength of 89 MW/cm^2^ and a light uniformity of 99% is used in the new multimaterial DLP 3D printing platform. These are the 2 most important printing settings for DLP 3D printing: slice thickness and single-layer exposure time. We used different settings for each material during the printing process, as shown in Table 1.

**Table 1.** Printing parameters of different materials used in this study

Resin type	Single layer exposure time	Slice thickness
Normal resin	6–11 s	0.05 or 0.1 mm
Active precursor	8–12 s	0.05 or 0.1 mm

### Selective electroless plating

The helmet parts manufactured by multimaterial DLP3D printing exist as a hybrid of normal resin/active precursor. The active precursor is obtained by doping Pd^2+^ ions into conventional resin. In order to achieve selective electroless copper plating on the surface of this composite material, we first soak the printed parts in a sodium hypophosphite (Na₂HPO₃) solution. The main purpose of this treatment process is to reduce Pd^2+^ ions to metallic Pd, thereby increasing its catalytic activity. The chemical reaction is described in Eq. (3).

$${\mathrm{Pd}}^{2+}+2{\mathrm{Na}}_{2}\mathrm{HP}O_{3}\to \mathrm{Pd}+2{\mathrm{Na}}^{2+}+2H_{2}O+H_{3}PO_{3}$$ (3)

After this reduction, the parts are immersed in an electroless copper plating solution for coating deposition. The presence of Pd makes the reduction process of copper ions (Cu^2+^) in the plating solution more efficient and ultimately forms a uniform copper coating on the surface of the active precursor, thereby achieving the desired conductive properties. The chemical plating bath composition and plating environment for electroless copper plating are shown in Table 2, while the chemical reaction can be summarized in Eq. (4).

$${\mathrm{Cu}}^{2+}+2e^{-}\overset{\mathrm{Pd}}{\to }\mathrm{Cu}$$ (4)

**Table 2.** Electroless Cu plating bath composition and operation conditions

Chemical	Concentration
${\text{CuSO}}_{4}\cdot 5H_{2}O$	50 g/l
${\mathrm{NH}}_{4}\mathrm{OH}$	1−2ml/l
HCHO	$10\%\left(v/v\right)$
$C_{6}H_{8}O_{7}$	10g/l
$H_{2}{\mathrm{SO}}_{4}$	pH adjustment
NaOH	
pH	10
Temperature	40 °C

### Testing and characterization

In order to test the effect of plating time on the surface resistance of parts, a series of samples were subjected to different copper plating times (with the same process parameters except for other parameters) and the corresponding surface resistance was measured. The samples were standard cuboids of 20 mm × 15 mm × 10 mm. The surface resistance was measured by GXMCP-T700 equipment, and the data were obtained by the 4-point probe method.

Our adhesion evaluation is conducted in accordance with the international standard ASTM D3359-09 (Standard Test Method for Measuring Adhesion by Tape Test). In order to test the effect of the main factor of adhesion (chemical etching time) on the adhesion of the metal layer, a batch of samples (other process parameters are exactly the same) was treated with different chemical etching times before electroless copper plating. The samples are standard cuboids of 10 mm × 10 mm × 8 mm. Table 3 shows the scoring criteria for the tape test. The adhesion of the resulting coating is evaluated by evaluating the metal coating adhered to the standard tape.

**Table 3.** Adhesion performance scale as per ASTMD 3359-02 standard

Classification	Percent of area removed	Surface of cross cut area from which flaking has occurred	
5B	0%	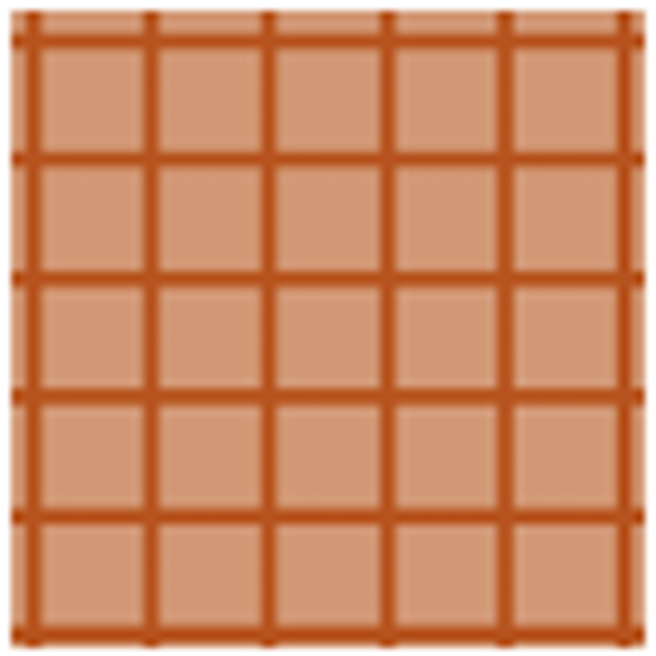	No metal came off after the tape adhered
4B	Less than 5%	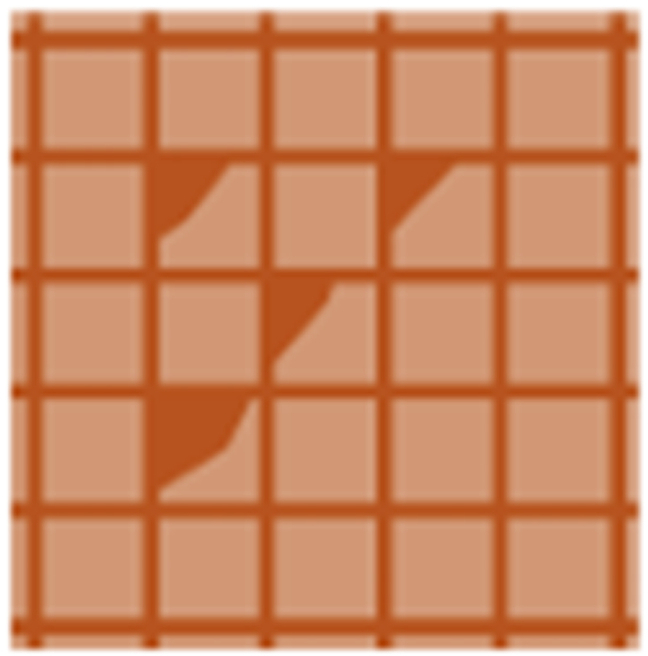	A very small amount (less than 5%) of the metal is adhered to and detached from the tape.
3B	5%–15%	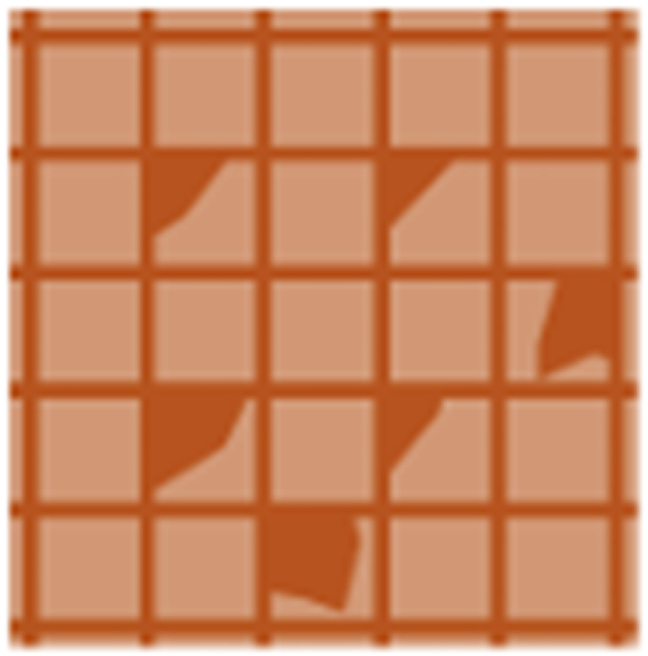	A relatively small amount (5%–15%) of the metal is removed from the taped.
2B	15%–35%	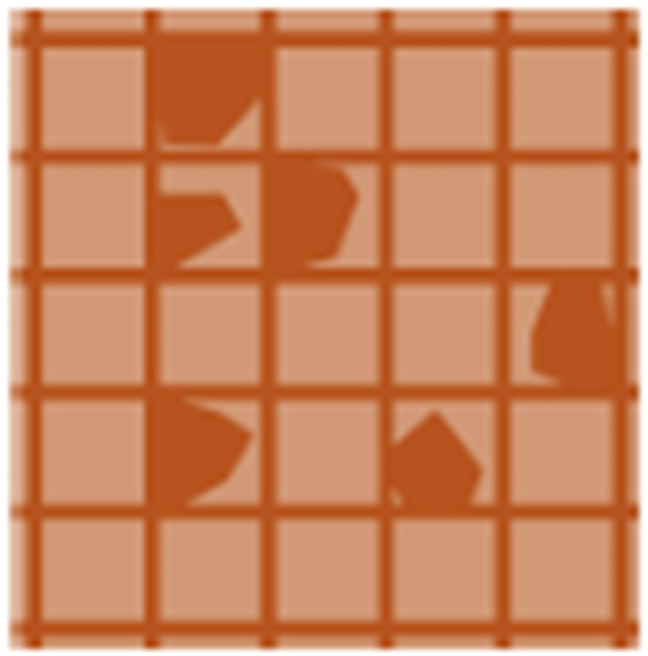	A relatively large amount (15%–35%) of the metal becomes detached from the tape.
1B	35%–65%	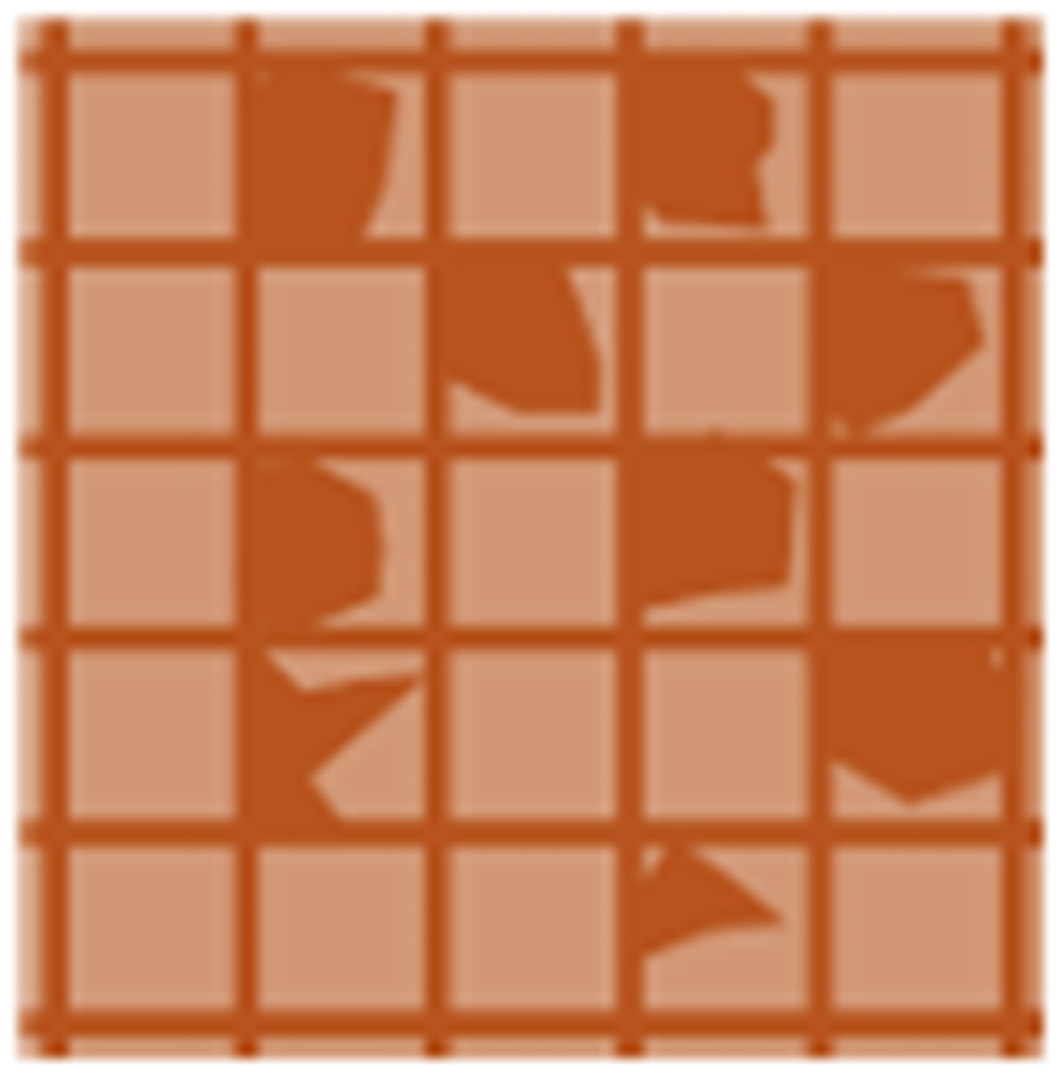	A significant amount (35%–65%) of the metal was removed from the tape.
0B	More than 65%	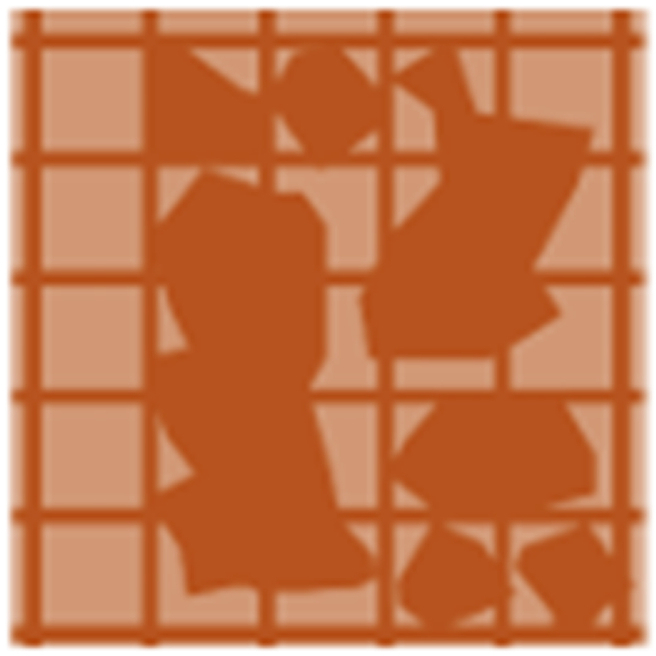	The metal plating is completely destroyed and there is a chance that all (more than 65%) of the metal plating is removed by the tape.

## Results and Discussion

### Wearable devices design

#### Wearable devices at the antenna

The scape was chosen as the electrode attachment site for antenna stimulation because of its beneficial characteristics. It is significantly larger than other parts of the antennae, maintains a nearly uniform diameter along its length, and serves as the connection point between the antennae and the head. We propose a reusable C-shaped cylindrical structure to simplify the attachment process. The elastic properties of the material allow the distance of the C-shaped groove to expand temporarily during the attachment process and return to its original position when the scape is completely inserted. The compression force between the inner wall of the C-shaped groove and the scape allows it to adhere firmly to the scape of the antennae. Therefore, this design simplifies the process of connecting the electrode to the antennae, such as eliminating the need to use adhesives to fix the electrode and allowing easy attachment and removal (Fig. 2A, ii and Fig. S1B).

To ensure the connection and relative position between the electrode and the antennae during navigation, a supporting structure is designed to fix the C-shaped cylindrical structure to the cockroach head. The head capsule, a hard chitinous exoskeleton, is used for connection. A hook mechanism is used to attach the support structure to the side and back of the head while avoiding damage to sensitive areas such as the compound eyes and mouthparts.

This design consists of 3 hooks forming a triangle: a central hook located at the top of the head and between the 2 compound eyes, and 2 symmetrical hooks placed at the bottom of the head capsule, one hook on each side of the head capsule near the mouth parts. This configuration equally spreads the clamping force over the head capsule. Furthermore, this design maintains the mouth part’s functionality so the cockroach may eat normally once it has attached to the wearable device (Fig. 2B).

The attachment process involves positioning the 2 lower hooks at the base of the head capsule first, followed by sliding the upper hook along the capsule’s surface. The material’s elasticity allows the upper hook to temporarily deform, expanding the distance between the hooks until it reaches the top edge of the head capsule. This allows the head’s top to slide into the hook wall. The supporting structure will be firmly fixed when all 3 hooks have contacted the side edge of the head capsule, ensuring the stability of the C-shaped cylinder during navigation (Fig. S1A).

The antennae will be stimulated electrically to control the cyborg insects. Thus, the C-shaped cylindrical structure and its surrounding parts are printed with an active precursor resin while the remaining parts are printed with normal resin. To ensure independent control of each antenna, the left and right sections of the structure are isolated. A groove separates these 2 sections, allowing independent electrical stimulation for the left and right antennae. Additionally, each section is designed with a block and cylinder hole to facilitate easier wire connections (Fig. 2A, iii).

#### Wearable devices at the abdominal segment

The conventional implantation methods involve the insertion of 2 electrodes on the second abdominal segment and cerci of the cockroach. However, the cercus is small and mobile, so it is hard to attach the electrode tightly. To address this issue, the sixth abdominal segment that is located nearby was selected as a replacement. This segment offers the advantage of an overlapping structure between the cockroach’s abdominal segments. The rigid exoskeleton and intersegmental membranes provide both flexibility and stability, ensuring reliable attachment without compromising the insect’s natural movement.

A wearable device was designed to attach securely to the second and sixth abdominal segments of the cockroach. The main structure of the device could be seen as a U-shaped clip, which closely touches the tergum dorsal and ventral at the same time to fix it along the *z*-axis (vertical direction). The rigidity of the cockroach’s exoskeleton at these segments forms a strong foundation for the device, anchoring it from vertical detachment during locomotion.

Important anatomical features of the tergum and surrounding segments were incorporated into the design to help prevent slipping along the edges of the tergum (*x*-axis). The curvature of the tergum creates a type of structural friction, and mechanically, the overlapped structure of the terga, where the posterior edge of one segment overlies the anterior edge of its neighbor and acts as a type of mechanical lock. This slot produced by overlapping terga is what the wearable device is designed to fit into to create anchoring with stability.

To eliminate sliding in the longitudinal direction (*y*-axis), we initially designed a hooking mechanism whereby the hooking parts have been designed with pointed edges and are angled for easy attachment at the edges of the cockroach’s abdomen segment (Fig. 2C, i). This pressure force was created because the gap between the upper and lower parts of the hooking mechanism was smaller than the thickness of the abdomen segment. Thus, the hooks are firmly held after attachment (Fig. 2C, ii). The disadvantage of this design is that it is not easy to fabricate even with the 3D printing method due to the small size of the groove and the smooth surface of the abdominal segments. To overcome these challenges, we modified the hooking mechanism to utilize hooks that clamp onto the edge of the tergum rather than relying on friction from pointed edges (Fig. 2D, i). This design is done with 2 hooks clamping the edge of a tergum. The 2 hooks attach symmetrically on either side of the extension so that force can be evenly distributed and the hooks can get a better grip on the curved edges of the tergum (Fig. 2D, ii).

This design integrates 3 main functions—the U-shaped clamp, the overlapping structure of the tergum, and the gripping hooks—which can cooperate to firmly fix the wearable device on the cockroach’s abdominal segment. The clip fits snugly along the natural contours of the cockroach’s body, providing high stability while allowing the insect to flex and extend its abdomen freely (Fig. 2E and Fig. S1C).

### Wearable device categorization and optimization

#### Wearable device at the antenna

The proposed wearable device as shown in Fig. 2C is developed from the 3D anatomical model of a standard cockroach head. The head size of cockroaches varies significantly from individual to individual and prevents the manufacture of a bespoke device for every specimen. To overcome this problem, 3 standard head types were established: small, medium, and large. These classifications were found by analyzing the data from a clustering method using the 2 most important dimensions of the head for a secure and functional attachment of the wearable device: the width and length.

A total of 3 distinct groups with characteristic dimensions derived from the clustering analysis are shown in Table 4. Individual head widths of cockroaches within the small group (*N* = 8 cockroaches) were 5.70 ± 0.20 mm, while head lengths were 6.10 ± 0.14 mm (*N* = 8 cockroaches). For the medium group, the average head width is 6.01 ± 0.18 mm, while the average head length is 6.57 ± 0.12 mm. On average, the large group has a head width of 6.39 ± 0.18 mm and a head length of 6.98 ± 0.15 mm (Fig. 2C). Compatibility across all relevant dimensions was then evaluated by analyzing additional parameters, including the distance between antennae and antenna diameter. Prototypes for the wearable device were designed based on the mean values of those 3 groups, respectively. For all prototypes, their corresponding group of smaller or larger head dimensions was designed to be within this mean value range.

**Table 4.** Summary of cockroach head and body dimensions by group

Group	Head width (mm)	Head length (mm)	Distance between antennae (mm)	Diameter of antennae (mm)	Cockroach length (mm)
Small	5.70 ± 0.20	6.10 ± 0.14	6.33 ± 0.30	0.94 ± 0.05	58.50 ± 2.71
Medium	6.01 ± 0.18	6.57 ± 0.12	6.78 ± 0.23	1.05 ± 0.07	61.00 ± 3.00
Large	6.39 ± 0.18	6.98 ± 0.15	7.23 ± 0.11	1.13 ± 0.05	67.43 ± 2.76

A one-way analysis of variance (ANOVA) confirmed a highly significant correlation between group classification and cockroach length (*P* < 0.0001), confirming the procedure performed to classify cockroaches into groups. This finding emphasizes the biological relevance of the classification and establishes its potential for standardizing wearables. The length of the cockroaches grew sequentially from the smallest group (58.50 ± 2.71 mm) to the moderate group (61.00 ± 3.00 mm) to the largest group (67.43 ± 2.76 mm), displaying the predictive correlation between head measurements and total body size.

Head dimensions were divided into 3 standard groups with applicable tolerances varying in width based on SD, allowing the wearable device design to comfortably embrace most individuals in each of the groups. This method, which balances individual spread to engineering of manufactured devices, serves as a strong paradigm for the design and fabrication of wearable devices in biological settings.

#### Wearable device at the abdomen segment

The abdominal hooking mechanism is less dependent on exacting sizing than that of the antennae, where it is critical that the 3-hook mechanism be fitted precisely to avoid swing when in use. Its design relies on 2 side hooks that lock onto the edge of the tergum.

In the observation of the cockroach in its standstill position, the overlapping lengths of Segments 1 and 2 and Segments 6 and 7 are 2.63 ± 0.2 and 1.69 ± 0.2 mm, respectively. A one-way ANOVA shows a highly significant difference of Segments 1 and 2 against Segments 6 and 7 (*P* = 8.58e−10). This indicates that the average gap sizes of Segments 1 and 2 and Segments 6 and 7 are significantly different. Hence, there is a valid reason for designing the abdominal wearable device in the second and sixth segments independently.

Intersegmental membranes ensure flexibility and are attached by intersegmental structures. These structures would enable the segments to flex and extend with the cockroach’s activities (such as running and breathing), inducing dynamic changes in the overlapping length. In particular, the length of overlap decreases when the cockroach stretches its body and increases when it contracts.

To account for these natural variations while keeping the cockroach’s physiological range of motion, the physical characteristics of the wearable device were determined using the longest observed overlap lengths: 2.9 mm (Segments 1 and 2) and 2.0 mm (Segments 6 and 7). This ensures a secure fit during all activities, from stationary positions to dynamic movements.

### Fabrication and performance of wearable devices using noninvasive electrodes with metal plating

We designed a highly adaptable (complex surface and size) 3D electronic wearable device for precise fitting on the cockroach’s head. The wearable device has a complex 3-dimensional structure and is selectively metalized in specific areas to achieve electrical stimulation. Due to the complexity of the wearable device’s shape, small size, and integration of electrical stimulation functions, it becomes challenging for traditional 2-dimensional electronic manufacturing processes and printed electronics technologies to meet the requirements of precise adaptation and functional integration. The combination of multimaterial DLP 3D printing method and selective electroless plating technology based on active precursors (photosensitive inks with catalytic activity) makes it possible to achieve high-precision functional manufacturing of 3D electronic devices [27], in terms of both functional integration [26] and microscale construction [33].

Fig. 3 and Fig. S2 show the conceptual process of our 3D electronic wearable device construction using multimaterial DLP 3D printing and selective electroless plating technologies based on active precursors, and its mechanical and electrical properties. As shown in Fig. 3A), the basic structure of the wearable device is first manufactured by multimaterial DLP 3D printing technology, which consists of hybrids composed of normal resin and active precursor groups with specific structures. Through this process, different materials are precisely positioned and printed to form different functional areas (metal cannot be deposited on the surface of normal resin, but active precursors can). Fig. 3B shows the evolution of material phases during manufacturing. In this process, the normal resin is composed of the photoinitiator, monomer, and prepolymer, but palladium ions (Pd^2+^) are added to the active precursor to make it catalytically active. Under ultraviolet (UV) light irradiation, both normal resin and active precursor form cross-linked structures to achieve the transformation from UV ink to 3D printed structure. In the active precursor structure, Pd^2+^ is embedded in the cross-linked network to generate precursor areas with metal plating attachment activity. These areas will be key in the subsequent selective copper plating process. The macro-molding process of the 3D electronic wearable device is shown in the upper left corner of Fig. 3A), which is achieved by multimaterial switching. After printing, the wearable device structure is cleaned with alcohol to remove excess uncured resin. Then, the structure is immersed in an electroless copper plating bath. Since areas with active precursors are introduced into some materials early, copper ions can selectively deposit in these areas to form metal coatings, such as the copper/plastic composite material structure shown in Fig. 3B. Through this composite manufacturing method, the wearable device contains conductive metal copper layers and insulating ordinary resin materials, thereby achieving functional partitioning and meeting electronic components’ implantation and connection requirements. Through this manufacturing method, the 3D electronic wearable device can have high-strength mechanical properties and integrate conductive paths, providing a hardware foundation for subsequent electrical stimulation to control cockroaches.

To ensure the functionality, mechanical performance, and electrical reliability of the 3D electronic wearable device, we tested its mechanical strength, coating structure parameters, and electrical performance through finite element analysis and electrical performance testing. This trend of surface resistivity variation with defined plating time is shown in Fig. 3C, i. With the increase of electroplating time, the surface conductivity of the coating first decreases and then increases. By comparing the surface resistivity in the first 40 to 50 min, the surface resistivity decreased significantly with the increase of electroplating time, which indicated that the gradual deposition of the copper coating played an important role in improving the conductivity, especially in 45 to 50 min, which reached the minimum resistivity, and the thickness and uniformity of the coating were optimal at this time. However, as shown by a new resistivity increase after more than 50 min, this may also be due to stress concentration or uneven deposition caused by the excessive thickness of the coating, which affects the conductivity. This phenomenon shows that if the electroplating time is too long, it will lead to a decrease in the quality of the coating. Thus, the electroplating time, which can balance both conductivity and coating degradation, should be 40 to 50 min by coupling with the conductivity side. This finding adds a crucial foothold for the further optimization of electroplating parameters. In addition, we also examined the tendency of adhesion level with coating thickness (plating time), presented in Fig. 3C, iii. The statistical analysis indicates a strong relationship between the thickness of the coating and the grade of adhesion. Against less thickness, in thinner coatings (1.23 to 2.06 μm), the adhesion grade is low, 1B to 2B, meaning that the adhesion of coating along the substrate is weak, which may be due to insufficient continuity in the deposition process. As the coating thickness is increased to 2.06 μm, the adhesion grade increases to 3B, however, reflecting that the thickness increases the adhesion between coating and substrate. It is also found that the adhesion grade reaches the maximum 4B and 3B in the medium thickness range of 2.4 and 2.67 μm. In this time, the thickness and quality of the coating are the most optimal, which obtains good adhesion performance. On the other hand, at thicknesses of 2.92 μm or greater, the adhesion grade decreases back to 2B to 3B either due to the weakening of the bond from high internal stress or due to the uneven thickness of the coating. We implemented a modification in chemical etching time to fulfill the requirement of adhesive grade, which appeared to be dependent on good conductivity.

Etching time is a complex parameter with an important influence on the adhesion grade, as illustrated in Fig. 3C, ii. In the first stage (10 to 20 min), the adhesion grade fluctuates from 2B to 3B, reflecting that the chemical etch is not effective in the short term. With the increase of etching time to 20 min, the adhesion grade of data 2 reaches 4B, demonstrating that etching treatment can remove the surface impurities and improve the adhesion of the coating. At the mid-stage etching (25 min), all data achieve the best adhesion state of 4B, contributing that this etching treatment removes surface defects moderately and enlarges the surface area. However, when the etching time reaches 30 min, adhesion starts to drop (2B), which is probably due to excessive etching of the substrate. In the late etching stage (40 to 60 min), data consistently showed poor adhesion (1B and 2B). However, at the 40-min mark, some samples temporarily surged to a higher adhesion grade (3B and 4B) before falling back to 1B and 2B at 45 min. This shows that excessive etching can destroy the integrity of the coating structure. Combined with the previous test results of coating thickness and adhesion, it can be found that when the coating thickness is between 2.4 and 2.67 μm, the adhesion reaches its optimal state. By comprehensive analysis of the above 3 experimental results, we confirm the ideal adhesion and conductivity of sample coating thickness and chemical etching time. The best electroplating time was found to be 40 to 50 min according to the surface resistivity test, with the coating thickness being at 2.4 to 2.67 μm here and surface resistivity reduced considerably, and the adhesion level at 3B to 4B is achieved between the coating and the substrate, meaning good bonding on a chemical level. Moreover, this new chemical etching process shows the greatest adhesion of 4B when the etching time is 20 to 25 min, which further improves the adhesion of the coating. With continuous etching, the adhesion has a downward wavy trend and decreases after 30 min, revealing that the long etching time would damage the substrate and weaken the coating structure. In conclusion, the best coating thickness is between 2.4 and 2.67 μm at 20 min to 25 min of chemical etching time. The coating can achieve excellent conductivity and strong adhesion under this condition, which provides an important reference for the overall performance optimization of 3D electronic wearable devices.

Under the above chemical etching time, we measured the curve of coating thickness changing with plating time, and the results are shown in Fig. 3C, iii. According to the analysis of the measured coating thickness data and plating time, the optimal target plating time should be 25 to 30 min. When the plating time is 25 min, the coating thickness is 2.4 μm, which has reached the lower limit of the best adhesion and conductivity. When the plating time is 30 min, the coating thickness increases to 2.67 μm, reaching the upper limit of the optimal range. Therefore, choosing a plating time of 25 or 30 min can ensure that the coating thickness meets the required optimal performance requirements, thereby providing a strong guarantee for the subsequent manufacture of 3D electronic wearable devices.

### Verification of wearable devices using noninvasive electrodes with metal plating

Firing rate analysis showed that neural activation in response to abdominal segment stimulation increased sharply with voltage (Fig. 4C, right panel). The firing rate rose from ~30 Hz at 1.0 V to over 200 Hz at 5.0 V, indicating strong and rapid neural excitation. Nevertheless, a wider SD band was observed at higher voltages, particularly beyond 3.0 V, indicating an increase in variability. This implies that while high-voltage stimulation effectively activates a greater number of neurons, it also introduces inconsistency in the response.

In contrast, antenna stimulation resulted in a more progressive increase in firing rate (Fig. 4C, left panel), with mean responses increasing steadily from 50 Hz at 1.0 V to approximately 140 Hz at 5.0 V. The increased response consistency and predictability across mid-range voltages (2.0 to 3.5 V) are indicative of a tighter SD, which is suitable for precision control during navigation.

These findings are reinforced by the CV analysis (Fig. 4D). The CV for the abdominal segment showed large fluctuations at low voltages (with extreme values >1 and even negative outliers), stabilizing only beyond 3.5 V. This high variability limits the usability of low-voltage stimulation for consistent motor control. In contrast, the CV for the antenna stimulation remained relatively low and stable between 1.5 and 3.5 V, dropping below 0.2 across this range. The lowest variability was observed at 2.0 V (CV ≈ 0.1), suggesting an optimal balance of activation strength and reliability.

The neural response of cockroaches was measured to examine the effectiveness of stimulation via wearable devices. Due to the nonlinear nature of the nervous system, the firing rate cannot be interpreted to the strength of animal reaction. However, these results provided useful insights regarding requirements for stimulus amplitude. Stimuli with small amplitudes elicited fewer neural spikes with a high CV. Such stimulation may lead to a low response rate of the animal, which was observed in the previous study using conventional implanted electrodes [17]. To induce animal reaction reliably, stimulation is required to produce stable neural reactions. On the other hand, maintaining stimulus amplitude below a certain level has a potential advantage on locomotion control. Li et al. [34] demonstrated that dynamic adjustment of stimulus amplitude enabled sustainable control of the cyborg insect for hours. In their study, the stimulation amplitude was increased or decreased with a fixed ratio when the animal’s reaction was below or above the threshold. Compared to stimulation with a fixed amplitude, cyborg insects walked continuously for 3 times longer with the dynamic stimulation amplitude.

After selecting the suitable voltages to stimulate the cyborg insects with the wearable devices, we evaluated the control performance by conducting experiments to control the cyborg insects’ turning and acceleration. The prestimulation linear mean was 23.81 (SD ± 15.75) mm/s, representing low movement with high variability. During stimulation, the mean linear velocity was significantly increased by 56.78 ± 20.86 mm/s (Fig. 5A). It can be quantified as this large change in linear velocity during stimulation, which shows that the wearable device served to accelerate the insect. The mean (SD) was 25.36°/s (±13.86°/s) before stimulation and 64.95°/s (±33.43°/s) during stimulation when making left turns, indicating significant stimulation effects (Fig. 5B). In the case of right turns, where the mean angular velocity before stimulation was 20.41°/s (±19.35°/s), the mean angular velocity during stimulation was 52.96°/s (±22.79°/s), proving that the wearable device could be utilized to perform right turns (Fig. 5C).

This method was shown to control turning and acceleration in the insect using noninvasive electrodes. Using previously validated stimulation types and intensities, stimulation was used to navigate the cyborg insect on an “S”-shaped pathway manually (Fig. 5D). The path was designed with 4 distinct segments: an entrance straight segment, a left turn, a right turn, and an exit straight segment. A total of 5 trials were conducted using 3 cyborg insects (*N* = 3, *n* = 5), with their trajectories tracked using a VICON motion capture system. To ensure stable and effective stimulation during navigation, electrode impedance was previously measured for all 3 cyborg insects. The sixth segment-to-ground pathway showed a mean impedance of 70.3 kΩ (SD = 6.2, CV = 8.8%), while the left and right antenna connections yielded 123.0 and 123.3 kΩ, respectively (CVs of 11.6% and 13.5%) (Table 5). These values confirmed good electrical contact and mechanical stability during operation. These impedance measurements demonstrate that the wearable system reliably maintained contact throughout experimental navigation.

**Table 5.** Impedance measurements (mean ± SD) and coefficient of variation (CV) for electrode pairs between the wearable device and cockroach body at 1 kHz

Electrode pair	Mean (kΩ)	SD (kΩ)	CV (%)
Sixth seg. → ground (second seg.)	70.3	6.2	8.8%
Left antenna → ground (second seg.)	123.0	14.2	11.6%
Right antenna → ground (second seg.)	123.3	16.6	13.5%

To quantitatively assess the spatial accuracy of cyborg insect navigation, we analyzed the deviation between the actual trajectories and the predefined path using the root-mean-square error (RMSE). Across the 5 trials, the RMSE values were 1.74, 1.80, 4.88, 3.30, and 2.82 cm, respectively, with a mean RMSE of 2.91 ± 1.17 cm, indicating that the insect could be guided with high spatial precision and repeatability using the proposed wearable system.

Stimulation commands during each trial were classified as “turn left”, “accelerate”, or “turn right”. On average, each trial contained 12.4 ± 7.4 left-turn commands, 16.6 ± 2.6 acceleration commands, and 14.2 ± 3.3 right-turn commands (Fig. 5E). Acceleration stimuli were issued most often because they kept the cyborg insect moving swiftly, especially along straight segments, thereby reducing overall traversal time. In one of the 5 trials, the insect initially drifted toward the left edge when entering the right-hand segment of the S-path (Fig. 5D). To re-center its trajectory, additional right-turn stimulations were applied in place of left-turn commands; consequently, that trial required only 4 left-turn commands (Fig. 5D). This outlier accounts for the larger SD observed in the left-turn category.

To characterize locomotion performance in detail, we analyzed the cyborg’s linear velocity for each segment of the S-path. The mean velocities for the entrance, left turn, right turn, and exit segments were 5.28 ± 1.91, 2.64 ± 0.84, 2.77 ± 0.57, and 4.73 ± 2.20 cm s^−1^, respectively (Fig. 5F). By comparison, adult *G. portentosa* cockroach walking freely—i.e., without electrical stimulation—move at roughly 3 cm s^−1^ [23]. Velocities fell below this baseline during the turning segments because stimulation was devoted to steering rather than propulsion, whereas they exceeded the baseline on straight sections where acceleration commands predominated. The significant velocity difference between accelerations and either turning or free walking means that the number of acceleration commands can vary the velocity over a wide range in the entrance and exit segments; consequently, these segments exhibit larger SDs.

Overall, these results demonstrate that the proposed wearable devices enable effective control of both direction and speed in cyborg insects for complex path-following tasks.

Moreover, in the real world, cyborg insects must be able to navigate landscapes with various obstacles. To validate obstacle negotiation performance, we performed experiments comparing the performance of devices with noninvasive electrodes taken to the body and devices with implanted electrodes. These experiments were conducted for 2 classes of obstacles—large obstacles (the large concrete blocks) and small obstacles (rocks) (Fig. S2B). Two metrics appeared for each of the evaluations: (a) time to traverse and (b) distance traveled.

With respect to small obstacles, connected devices also had faster mean crossing times (15.45 ± 8.90 s vs. 48.56 ± 30.45 s—*P* < 0.05 for the implanted electrodes). However, this difference was not statistically significant (*P* = 0.07527). Wearable devices had a mean distance of 450.67 ± 150.23 mm, which was shorter than the mean distance of 900.32 ± 310.78 mm traveled by implanted electrodes, though this difference was also not statistically significant (*P* = 0.11747) (Fig. 6B and Table 6). For smaller obstacles, this implies that the rocks’ circular (cylindrical) form can be advantageous to the cyborg insect in that it can navigate walking around the outside of the rocks or at the vertices of 3 rocks jammed into positions against themselves. Because the rocks are relatively low, the cyborg insect can scale down the rock surface’s slope after trying several times to get around and hit the floor (Fig. 6C, iii and iv).

**Table 6.** Mean, standard deviation, and *t* test *P* values for the duration and distance of wearable devices and implantation methods over large and small obstacles

Size of obstacles	Methods	Duration (s)		Distance (mm)		*t* test *P* value	
		Mean	SD	Mean	SD	Duration	Distance
Large	Wearable devices	23.16	14.34	789.34	310.45	0.01107	0.00140
	Implantation	78.25	45.12	1,745.89	520.32		
Small	Wearable devices	15.45	8.9	450.67	150.23	0.07527	0.11747
	Implantation	48.56	30.45	900.32	310.78		

However, in 3 trials in which the cockroach was fitted with implanted electrodes, the insect ascended the rocks but continued walking, leaving its torso unangled and not in contact with the floor (Fig. 6C, iv). The opposite was seen in the case of the wearable device (Fig. 6C, iii). In this configuration, the intact antennae allow the insect to sense the surface at these locations, triggering its decision to climb down. The antennae also retain their functional integrity in the wearable device, enabling the cyborg insect to employ its natural biological sensory organ’s relative navigational precision, thus further validating the added advantage of the wearable device in executing obstacle negotiation.

Wearable devices performed significantly better in navigating large obstacles than implanted electrodes. Wearable devices had a significantly lower mean traversal time (23.16 ± 14.34 s), shorter than implanted electrodes (78.25 ± 45.12 s, *P* = 0.01107). In the same way, the indentation distance of wearable devices (789.34 ± 310.45 mm) was significantly shorter than that recorded for implanted electrodes (1,745.89 ± 520.32 mm, *P* = 0.00140) (Fig. 6B and Table 6). The improved performance of wearable devices on large obstacles can be attributed to the relationship of the cyborg insect with its environment. Big obstacles with flat surfaces and sharp corners seem to confuse the cyborg insect with cutting and fixing antennae. They hesitate to descend the inclined plane, remaining on the upper surface and along the periphery. Moreover, they briefly explore downward on vertical walls before climbing back up, as they are unable to locate a horizontal surface, such as the ground (Fig. 6A, ii). This phenomenon increases both the distance and the time required for travel.

In contrast, the headgear allows the cyborg insect to use its antennae naturally. The antennae are about 35 mm long and will provide better information about the obstacles [24,35]. When the antennae brush the ground while the insect is on a wall, they trigger an immediate downward pivot, letting the cockroach leave the obstacle quickly.

## Conclusion

This research introduces novel ergonomic wearable devices featuring noninvasive electrodes and metal plating, addressing critical challenges in current cyborg insect technologies. The ergonomic devices comprise hooking mechanisms that are designed to firmly attach to the cockroach’s body, such as the antenna’s scape, the head, and the abdominal tergum. Thus, the headgear and abdominal buckle do not require adhesive, which means that they can attach and detach quickly, significantly reducing the preparation time and allowing the reuse of these wearable devices. These devices were manufactured via advanced manufacturing techniques (e.g., multimaterial DLP 3D printing and selective electroless plating) with high structural resolution and reliable electrical performance. By using these ergonomic wearable devices, the cyborg insect can successfully navigate a predetermined “S”-shaped path. Furthermore, the experiment highlights the critical role of antennae in obstacle navigation and underscores the necessity of the wearable device. This work paves the way for scalable, efficient, and innovative applications of cyborg insects in robotics and biohybrid systems.

Building on these findings, future work will focus on 2 priorities that improve scalability and navigation ability. First, our objective is to determine the minimum viable body size for the wearable system to function effectively. Because the tri-hook headgear and the C-shaped scape connector both rely on elastic fastening for attachment, their ability to attach is reduced below specific morphological thresholds. In order to resolve this issue, we will (a) parametrically downscale the mechanical design, (b) conduct systematic pull-off force measurements using progressively smaller cockroach models, and (c) determine the minimum body length and antennal scape diameter that still guarantee a retention force for stable engagement. Secondly, we intend to transition from manual to closed-loop autonomous control by incorporating an integrated inertial measurement unit (IMU) into the electronic backpack circuit. Real-time feedback on behavioral states, including ascending, pausing, and becoming stuck, will be provided by the IMU. The cyborg insect will be able to autonomously navigate using its preserved antennae by triggering context-aware control logic, such as halting turning stimuli when an obstacle is detected. The objective of this self-regulating strategy is to improve the robustness and autonomy of cyborg insect navigation in real-world environments.

## Data Availability

The data that support the plots in this paper and other findings of this study are available from the corresponding author upon reasonable request.
