# Supplementary material for: Ergonomic Insect Headgear and Abdominal Buckle with Surface Stimulators Manufactured via Multimaterial 3D Printing: Snap-and-Secure Installation of Noninvasive Sensory Stimulators for Cyborg Insects
Source: Cyborg Bionic Syst. 2025 Sep 22;6:0406. doi: 10.34133/cbsystems.0406 (PMC12451109; doi:10.34133/cbsystems.0406)
Supplement: Supplementary 1 — Figs. S1 to S3 Movie S1 Data S1 and S2 [file cbsystems.0406.f1.zip › Manuscript_Clean version.docx]

SUPPLEMENTARY MATERIALS


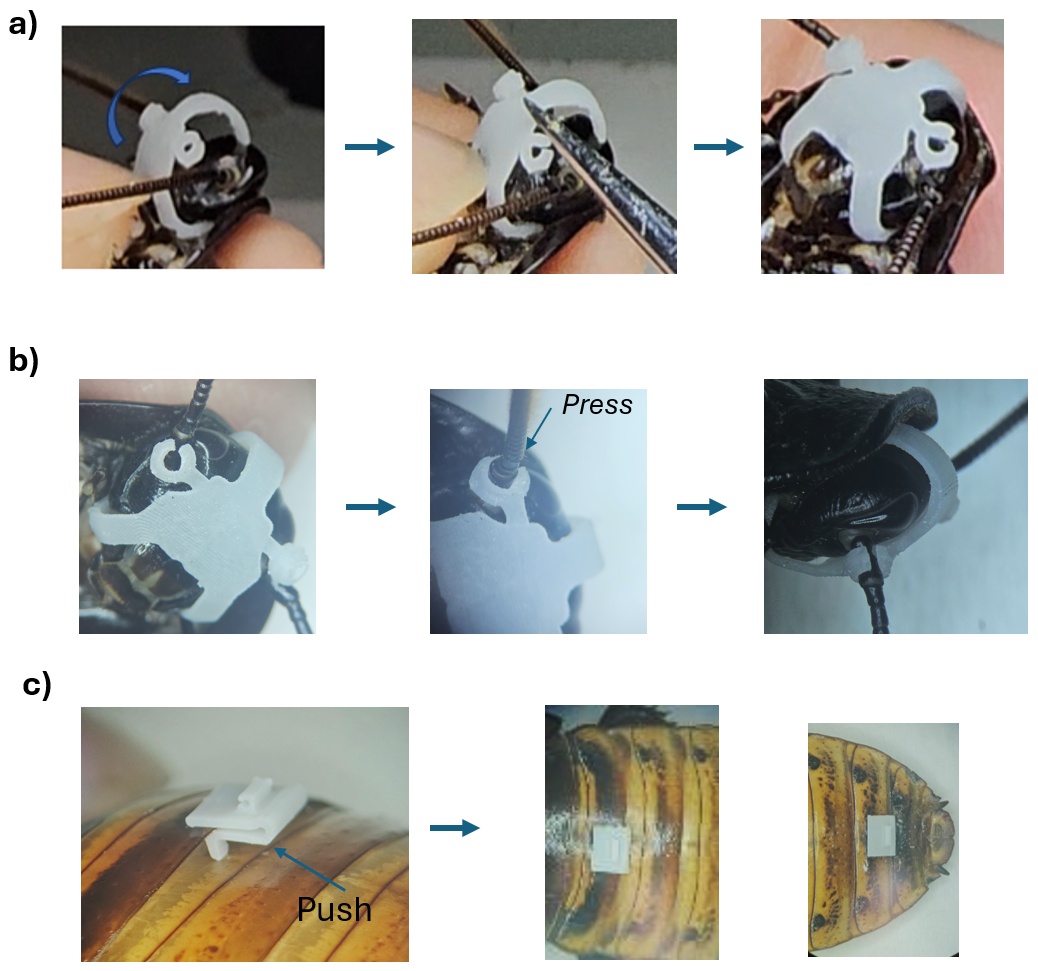


**Figure S1. Procedure to Mount the Wearable Devices to the Cockroach Body.** **a)** The two lower hooks of the wearable device were positioned beneath the cockroach’s head. The top hook was gently placed at the top of the head using tweezers, allowing its elastic material to stretch and temporarily deform to fit securely along the sides and partially onto the back of the head. **b)** The antenna was slid into the gap in the C-shaped connector and pushed inwards. The elasticity property of the connector made the components fit tightly. A thin layer of conductive paste (Spectra360, PARKER LABORATORIES, USA) applied to the inner surface of the connector ensured stable electrical contact with each antennae scape, allowing for reliable stimulation. The same steps were taken for the second antenna. **c)** The grounding was done via the second abdominal segment. The lower hooks of the omnidirectional device were placed in the clefts between the 1st and 2nd, as well as the 6th and 7th abdominal segments, holding firmly onto the edges of the tergum to maintain a stable electrical interface.


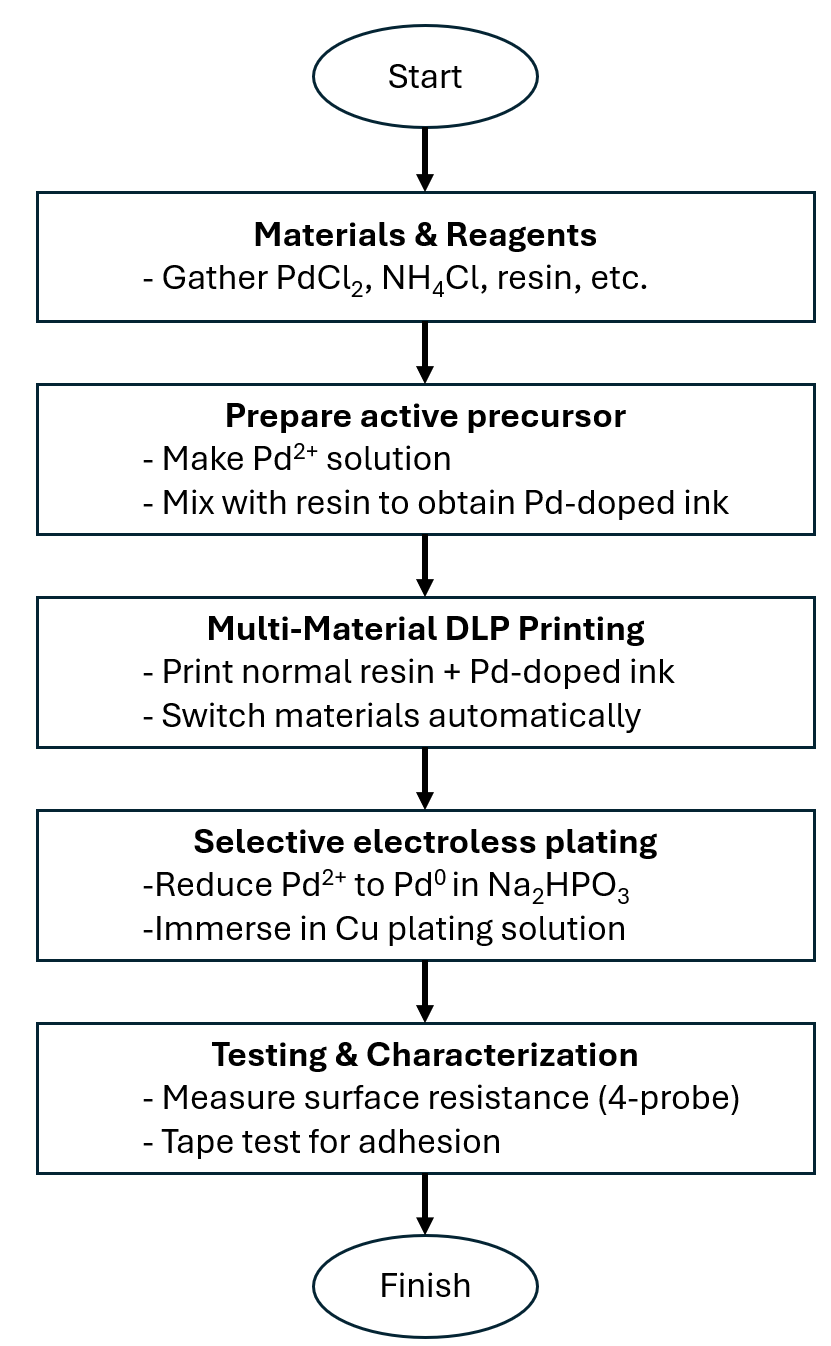


**Fig. S2. Schematic flowchart illustrating the five key steps for fabricating and characterising the copper-plated wearable device.** In Step 1, the raw materials (*PdCl₂, NH₄Cl, resin*, etc.) were gathered. Step 2 prepares the Pd-doped active precursor ink. Step 3 uses multi-material DLP 3D printing with normal and Pd-doped resin. Step 4 applies the selective electroless plating, and Step 5 concludes with measuring surface resistance (using the four-probe method) and conducting a tape test for adhesion.


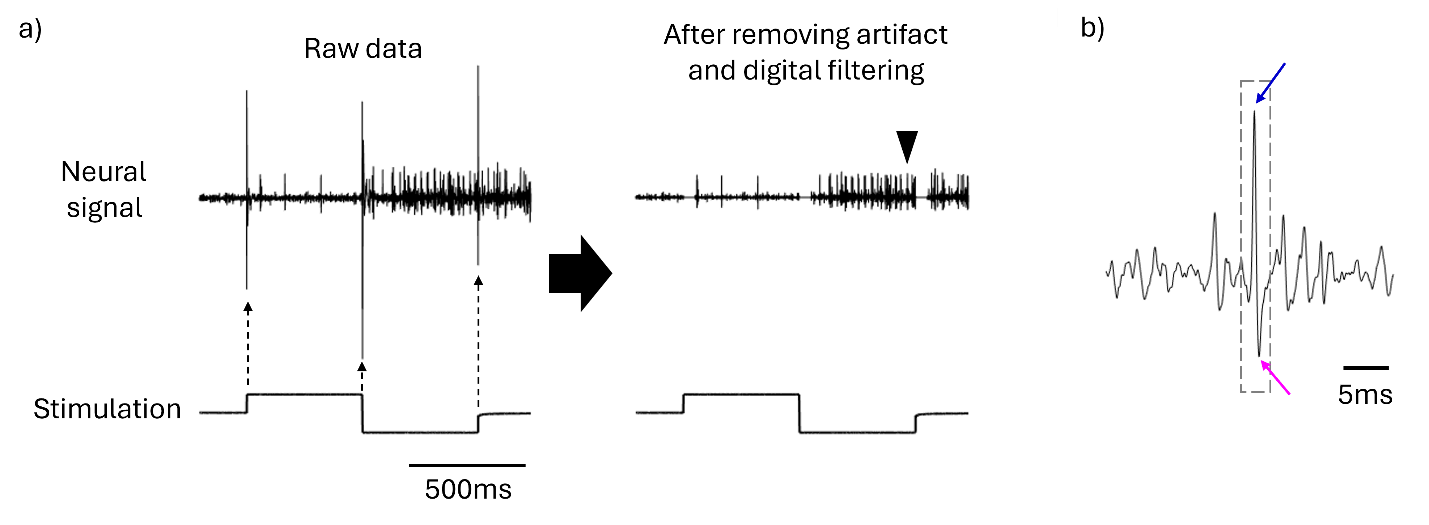


**Fig. S3. Neural signal and analysis. a)** Preprocessing of neural signal. Raw data included artifacts at the rising and falling edge of the stimulation pulse (arrows, Left panel). After replacing data in 50 ms window at the edge of the pulse with zero, neural signal was digitally filtered for spike detection (Right panel). Arrowhead indicates a single neural spike magnified in b. **b)** Waveform of neural spike. A neural spike (dashed line) showed single positive peak (blue arrow) and following negative peak (magenta arrow).

**Video S1.** “S” Path Navigation Demonstration of cyborg insect equipped with wearable devices. We manually controlled the cyborg insect along an approximately 1m long “S”-shaped path.

Data S1. STL file of the small obstacle 3D CAD model used in the experiment.

Data S2. STL file of the large obstacle 3D CAD model used in the experiment.
